# Analysis of Multiple-Access Discrimination Techniques for the Development of a PSD-Based VLP System

**DOI:** 10.3390/s20061717

**Published:** 2020-03-19

**Authors:** Álvaro De-La-Llana-Calvo, José-Luis Lázaro-Galilea, Alfredo Gardel-Vicente, David Rodríguez-Navarro, Borja Rubiano-Muriel, Ignacio Bravo-Muñoz

**Affiliations:** Department of Electronics, University of Alcalá, Alcalá de Henares, 28801 Madrid, Spainalfredo.gardel@uah.es (A.G.-V.); david.rodriguezn@uah.es (D.R.-N.); ignacio.bravo@uah.es (I.B.-M.)

**Keywords:** Visible Light Positioning (VLP), PSD sensor, multiagent, CDMA, FDMA, optical signal

## Abstract

There are several technologies and techniques available when developing indoor positioning systems (IPS). Recently, the development of positioning systems based on optical signals has aroused great interest, mainly those using visible light from the lighting infrastructure. In this work, we analyze which techniques give better results to lay the foundations for the development of a Visible Light Positioning system (VLP). Working only with a receiver, it is analyzed what the result of determining the position of different emitters is when they emit simultaneously and without any synchronism. The results obtained by Frequency Division Multiple Access (FDMA) (with digital bandpass filters, I/Q demodulation, and FFT) and Code Division Multiple Access (CDMA) are compared. The interference between signals when emitted simultaneously from multiple emitters is analyzed as well as the errors they cause and how these effects can be mitigated. As a result of the research, the advantages and disadvantages using different multiple-access determination techniques are determined. In addition, advantages and disadvantages of using FDMA and CDMA techniques as well as hardware requirements that make one more feasible than the other are presented. The system behavior, in terms of errors, is established using FDMA and different configurations such as: I/Q, RMS, or FFT. The work also determines the error rates that can be obtained with the different FDMA and CDMA configurations, considering different error scenarios and integration time. Synthetic emulations and empirical tests were performed, which concluded that IPS systems based on optical signals and PSD sensors can achieve very high measurement accuracies and a high measurement rate. Obtained positioning errors in a room of 3 m height are less than 1 cm when working in noisy environments.

## 1. Introduction

Different applications have been developed based on Indoor Positioning Systems (IPS) to assist people [1] to locate users in large indoor environments in both professional and leisure activities [2,3]. They are also used in logistic applications for intelligent factories [4] to perform different tasks such as moving objects within an environment [5].

It can be seen that IPS is already well established in daily activities [6,7,8,9]. It is a fact that, knowing the user position in indoor activities, provides new capacities to applications with significant added value.

In indoor positioning, no technology has been imposed as the GPS has in outdoor positioning. By contrast, different techniques are complementary and cooperative, while the characteristics of the environment itself limit the use of one particular technology.

Taking into consideration the intended application for which an IPS system is going to be used, power consumption, coverage, accuracy, privacy, and cost are key factors to select the technology to use [10,11,12].

Our paper is related to IPS based on the optical signal. In this regard, several research works make use of LED lighting, sharing the cost of the installation for illumination. The authors of [13,14] obtained positioning data relevant to compare accuracy and precision.

In [13], each LED in a lighting sends 3D space coordinates and an image sensor receives the signal. By numerical analyses, the authors showed that, when using a 1000×1000 pixels image sensor as receiver, the receiver’s position can be measured with an accuracy error of less than 1.5 m. In [14], the authors used a high-speed complementary metal-oxide-semiconductor image sensor to receive the digitized information. The sampling frequency of the image sensor is up to 48 kHz. The LED lights send information frames containing a prefix, ID, and Cyclic Redundancy Check (CRC) code.

The receiver obtains the world coordinates of the LED light from the received ID. In addition, they implemented a self-location estimation program using the Levenberg–Marquardt algorithm and conducted a positioning experiment using a fixed fish-eye camera and LED lights. The test area measured 5.4 m by 7.5 m, and the ceiling height was 3 m. The results show that the maximum horizontal error was 10 cm.

Huynh and Yoo [15] proposed the use of four LEDs that transmit the tridimensional coordinates getting positioning errors below 10 cm, improving the results given in other works [13,14].

Other articles (e.g., [16]) foster the development of IPS based on received signal strength (RSS) of the visible light from LEDs, using multiple photodiodes in the receivers. The results obtained show an average positioning error of 20 cm in an effective positioning range of 1.8 m. It should be noted that these systems have the handicap of SNR attenuation with greater distances and angles. Zachar et al. [17] proposed systems with infrared (IRED) emitters and IRED cameras, optimized for the detection of IRED beacons.

A system that determines the position by triangulation is presented in [18]; it emits IRED signals from the mobile agents and determines the angle of arrival with a set of photodiodes (detectors) in the environment. The test area was 7 m × 2 m, obtaining errors up to 70 cm. The handicap of this system is the difficulty of initial deployment and calibration.

Concerning the determination of several agents simultaneously, an orthogonal frequency division multiplexing (OFDMA) based system is proposed in [19]; the receiver recovers all the transmitted signals using discrete Fourier Transform (DFT) obtaining an average error of 1.68 cm and exceeding the maximum error of 2 cm. In this case, the area used in tests was small.

Concerning the IPS detector proposed in this paper, there are some research works using PSD (Position Sensitive Detector) sensors. In [20], the authors proposed a PSD-based IPS with a Kalman filter to track the agent moving in a plane surface. Error of location reached a maximum value of 8.97 cm and an average of 1.97 cm.

The work in [21] presents an alternative location system, which is based on image processing and performs most of the processing analogically. The location sensor is based on a PSD to locate the agent by trilateration. In the work, practical tests were carried out with a marginal disturbance of ambient light. A light lamp was placed at a height of 20 m and moved laterally, in a straight line, from a position above the origin (sensor location) to a distance of 6 m. At distances of displacement of less than 70 cm and more than 440 cm, it was observed that the measurements presented alignments and errors greater than in the rest of the range, where the behavior was linear. The positioning errors for distances over 440 cm were over 150 cm while between 70 and 440 cm the errors were barely 10 cm.

The authors indicated that the reasons for observable non-linearity are unknown. It seems quite plausible that the lens system, the operational amplifiers, and/or the position-sensitive device were the causes of these non-linearities. They indicated other digital processing steps might be able to compensate for these non-linearities.

## 2. Background

Our research group has worked in the development of optical IPS systems for more than a decade, carrying out work based on measuring the phase shift of the signal on arrival at detectors and determining the angle of arrival of the signal.

**Figure 1:**
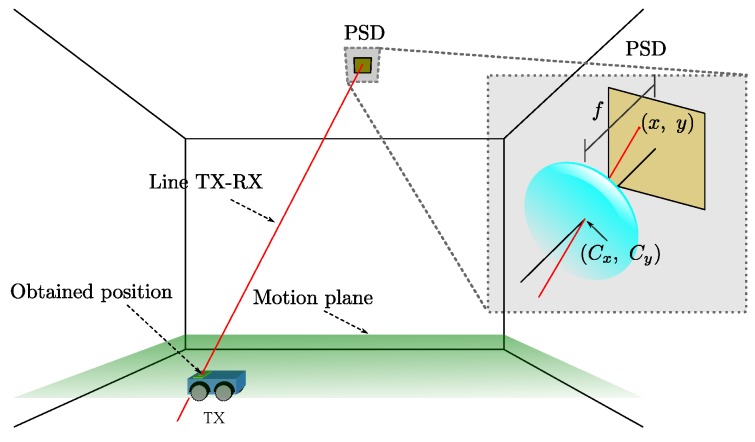
Diagram of the positioning system based on PSD sensor.

Focusing in IPS based on PSD, we have proposed an IPS [22] based on the angle of arrival (AoA) where we show some preliminary results of such a system. The following is an outline of the IPS proposed in [22]: knowing the impact point on the PSD, we can obtain the equation of the LOS to the emitter from the calibrated optical system. Knowing this line and knowing that our emitter is always moving in the same plane, the position of the emitter is given by the intersection of this line with that plane. A diagram of the positioning system can be seen in Figure 1.

The main advantages of IPS based on PSD sensor are accuracy, precision, range, and obtaining the impact point on the sensor surface with continuous resolution.

The main disadvantages in the development of IPS systems based on the optical signal are related to multipath effect due to NoLOS signals and distortions of the PSD and lens.

Regarding the distortion, in [23], the sources of error due to electronic components were studied in a PSD-based system, providing a solution to correct these errors. In [24], the geometric model and calibration of an IPS PSD-based system were developed.

On the other hand, joint-effort research has been done on optical signal reflection models to try to quantify and measure the effect of multipath on the development of IPS. The optical signal reflection model reported in [25] allows modeling how the multipath affects the measurement techniques based on AoA and PoA. In [26], a procedure was proposed to measure the signal value that impacts into the PSD (detector) surface as a result of MP. In [27], position errors due to MP were characterized by working with AoA and PoA techniques, to complement the previous works.

In addition, it has been studied which technique is more optimal to use in multipath environments using the PSD as a detector [27]. As a result, it was concluded that, with the use of AoA and being careful in the deployment of the LED emitters and focal length of the sensor optics, the results were much better than using phase differential of arrival (PDoA) [28,29,30] since the multipath received were compensated partially given the principle of function of the PSD.

Considering those background results as initial conditions, in this paper, we study the positioning of an agent that incorporates a single PSD, which receives the signal from several LEDs used for lighting. The problem to be solved is the error-free discrimination of the AoA received from each of the emitters, without affecting the reception of other signals simultaneously. Note that the PSD is a continuous sensor that generates currents proportional to the strength of the signals it receives and the impact point on its surface. Therefore, multiple signals from different emitters might affect the positioning as currents corresponding to each emitter will be mixed, generating a single output current.

De Lausnay et al. [31] gave an overview of the current state-of-the-art studies on visible light positioning systems classified according to the multiple access technology use. They showed the results for the sensors commonly used in positioning, such as photodiodes or image sensors. Regarding photodiodes, they showed the advantages and disadvantages of the use of Time Division Multiple Access (TDMA), Frequency Division Multiple Access (FDMA), or Code Division Multiple Access (CDMA), which are mainly used with PoA or ToA proposals. With respect to image sensors, they only mentioned Spatial Division Multiple Access (SDMA), because image sensors have a low sampling rate. VLP based on image sensors does not use TDMA, FDMA, or CDMA because the frame rates of smartphones cameras range from 30 to 120 fps, which is below the required values. In our case, the PSD sensor is a photodiode that continually obtains the impact point on the sensor surface from the light of a emitter. This allows us to use AoA techniques with high frequencies (i.e., 100 kHz), thus TDMA, FDMA, and CDMA techniques can be used.

In this work, several multiple-access discrimination techniques for the development of a PSD-based VLP system are analyzed using different types of signals and signal processing. It is more convenient to use these techniques to receive several signals simultaneously and without synchronism in a single detector, to be able to discriminate the AoA of each one of them with the minimum possible error.

The behavior of each technique is analyzed in different scenarios, varying the test configuration, the SNR, the number of simultaneous emitters, etc. Experimental tests with real signals were carried out. The goal of the empirical systems is to work with different signals received simultaneously.

## 3. Signal Determination Techniques in a Single Detector Based LPS

**Figure 2:**
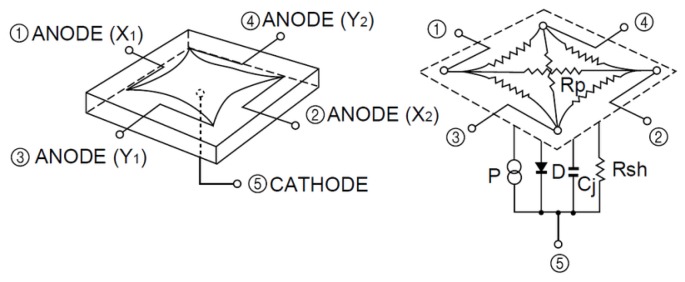
Equivalent circuit of the Position Sensitive Device (PSD) pin-cushion (image courtesy of Hamamatsu, obtained from the PSD technical information).

The PSD sensor allows us to calculate the signal reception point on the PSD surface based on the four electric currents generated at each PSD anode. PSD sensor is a type of photodiode that consists of four anodes and a common cathode. Figure 2 shows the model of a pin-cushion two-dimensional PSD.

The PSD sensor allows us to calculate the signal reception point on the PSD surface based on the four electric currents generated at each PSD anode according to Equations (1) and (2)

(1) $$\begin{array}{c} x=\frac{L_{X}}{2}\frac{\left(I_{X2}+I_{Y1}\right)-\left(I_{X1}+I_{Y2}\right)}{I_{X1}+I_{X2}+I_{Y1}+I_{Y2}}, \end{array}$$

(2) $$\begin{array}{c} y=\frac{L_{Y}}{2}\frac{\left(I_{X2}+I_{Y2}\right)-\left(I_{X1}+I_{Y1}\right)}{I_{X1}+I_{X2}+I_{Y1}+I_{Y2}}, \end{array}$$

where $I_{X1},I_{X2},I_{Y1},$ and $I_{Y2}$ are the electrical currents from the PSD sensor anode pins and $L_{X}$, *y*, $L_{Y}$ are the sensor dimensions.

The aim here is to obtain the signal reception point on the PSD surface of each of the emitters. Different techniques that can be used for this purpose are analyzed below, choosing the most suitable one for the empirical tests.

In the next sections, we analyze FDMA and CDMA techniques used in IPS based on PSD. The use of TDMA has been ruled out due to it needs for synchronism between the emitters and the receiver.

### 3.1. Frequency-Division Multiple Access (FDMA)

**Figure 3:**
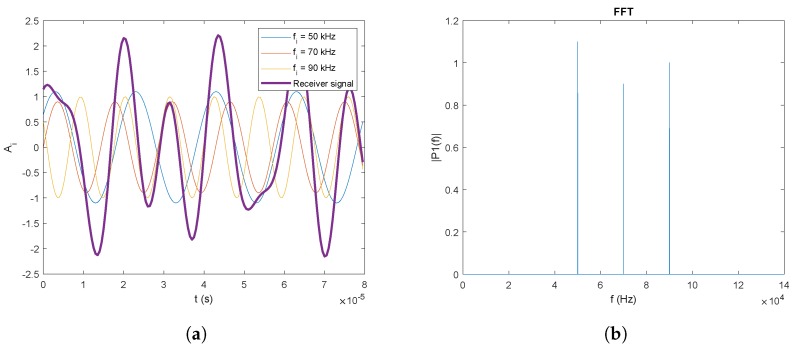
Example of three emitters using FDMA: (**a**) in the time domain; and (**b**) in the frequency domain.

When implementing this technique in a real system, there are some aspect that must be taken into account. From the example shown in Figure 3a,b, with ideal signals generated by computer, it seems at first sight that it would be easy to separate the signal of each of the emitters. However, in real systems, the signal from the emitters will not be a perfect delta, but, depending on the design of the emitter, that delta will have a significant frequency bandwidth, and harmonics will appear at multiples of the center frequency. On the other hand, the bandwidth of the PSD sensor is not infinite, but behaves as an order 1 low-pass filter, with a surface area that fixed the cut-off frequency (in the case of a 9×9 mm${}^{2}$ PSD, the bandwidth is around 200 kHz). The detection of each emission must be discriminated from the rest of emitters avoiding interferences among emitted signals. In FDMA, each emitter uses a different frequency signal. Therefore, the BW must be divided by the number of different frequencies, which in turn limits the number of emitters.

When choosing the different frequencies to be used by each of the emitters, the following aspects must be taken into account:

- The number of available frequencies depends on the width of the filter that can be implemented. The narrower is the filter, the more frequencies can be used, but the delay and the complexity of implementing the filter in a real system increase.
- frequencies that are not multiple of others should be chosen to avoid, as much as possible, interferences due to harmonics.
- The bandwidth of the emitted signal should not overlap with emitters on adjacent frequencies.

After knowing the limitations of the number of simultaneous emitters, the next step is to know how to implement the detection of each emitter. The objective is to know the root mean square (RMS) value of each emitter for each of the four channels of the PSD. The RMS value is used to avoid the influence of noise, which can be considered as white Gaussian noise with a null average. If the RMS value is obtained for a certain amount of signal, the noise is averaged and its effect is compensated as far as possible. The longer is the averaging time, the smaller is the noise influence, but the longer it takes to obtain a new measurement. Once the four values RMS have been obtained, the point of incidence on the surface of the PSD of the emitted signal by each of the emitters can be obtained.

Next, we propose three different methods to obtain the RMS value of each emitter.

#### 3.1.1. Discrimination Using Band-Pass Filters

This technique consists in the implementation of a bandpass filter for each of the system’s emitters. The signal received by each channel of the PSD, $m_{t}\left(t\right)$, is passed through a bank of filters. The output of each filter corresponds to the signal filtered for each one of the frequencies of the emitters. Once the filtered signal is obtained, some signal periods are used to calculate the RMS value. The number of cycles will be a function of the signal-to-noise ratio available.

**Figure 4:**
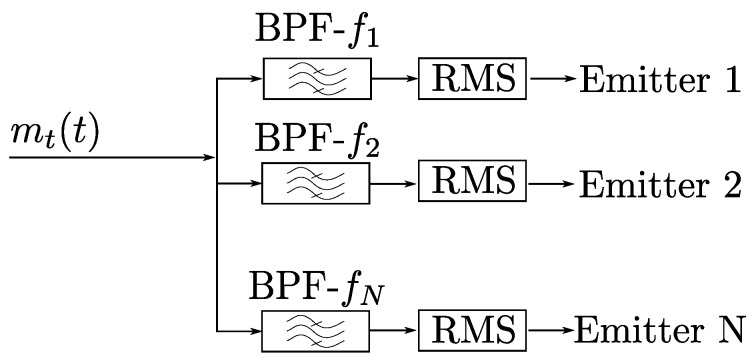
Bandpass filter diagram of one of the channels.

A diagram of this technique for each channel of the PSD can be seen in Figure 4.

With this technique, it would be necessary to design and implement four equal bandpass filters (BPF) (one filter for each channel of the PSD) for each emitter of the positioning system.

To obtain the RMS value of each emitted signal, it would be necessary to wait to acquire an integer number of periods of that signal. However, if the number of periods is high enough, the difference between using an integer number of periods or taking a part of the final period is insignificant. This method is useful to be able to use a fixed number of samples for all the emitters without having to change the number of samples for each emitter.

#### 3.1.2. Discrimination Using FFT

From the Discrete Fourier Transform (DFT) of the signal of each of the four channels of the PSD, it is possible to discriminate between the emitters of the LPS and to obtain the signal reception point on the PSD surface for each one of them. To obtain the DFT of the signal, the Fast Fourier Transform (FFT) algorithm is used. Once the FFT of the signal for each channel has been calculated, the sample modules corresponding to the frequencies of the emitters are obtained. For example, in Figure 3b, one can clearly see in the FFT the modules of the samples that correspond to the frequencies of 50, 70, and 90 kHz.

Depending on the sampling frequency and acquisition time, the bandwidth of each FFT sample will be different:

(3) $$\Delta f=\frac{f_{s}}{L}$$

where *L* is the total number of samples of the signal. The longer is the acquisition time, the lower is the Δf, and therefore less noise is “introduced” into the desired sample.

The sample $k_{i}$ for the frequency $f_{i}$ of the emitter *i* is calculated:

(4) $$k_{i}=\frac{f_{i}}{\Delta f}$$

This technique is especially suitable for microprocessor-based systems (sequential execution) because it allows discriminating a high number of emitters by performing only four FFT, one for each channel of the PSD, without the need to implement four filters for each emitter. One of the disadvantages is the need to take a high acquisition time to have a narrow bandwidth. In the case where the signal from the emitter is not a delta or a higher bandwidth is needed, more than one sample can be used around the desired frequency.

#### 3.1.3. Discrimination Using IQ Demodulator

Using an IQ demodulation, it is possible to obtain, from the received signal, the module and the phase of each of the signals of the system’s emitters. Each received signal is multiplied by a sinusoidal signal and by the same sinusoidal signal displaced π/2 rad, for each of the frequencies used by the emitters, obtaining the signals in-phase I(t) and quadrature Q(t). Each of these signals is passed through a low-pass filter (LPF) to obtain only the low-frequency components. The module or RMS value is calculated with that resulting signal.

**Figure 5:**
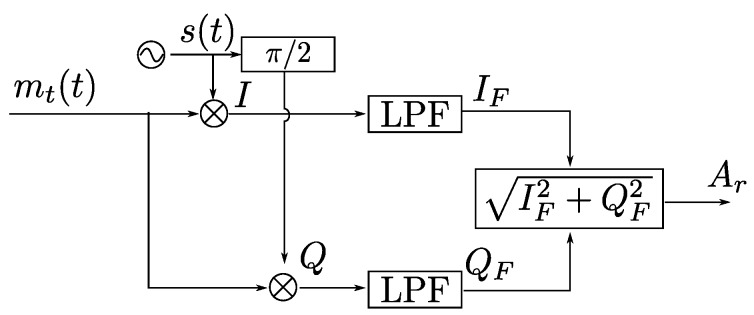
Diagram of an IQ demodulator.

An overview of the IQ demodulator is shown in Figure 5.

The received signal is $m_{t}\left(t\right)$ and s(t) is the sinusoidal signal frequency $f_{i}$. The LPF blocks correspond to low-pass filters to obtain only the low-frequency information.

In Equation (5), the equation of the received signal when only the signal from an emitter is received in the absence of noise is shown.

(5) $$m_{t}\left(t\right)=A_{i}\sin\left(2\pi f_{i}t+\phi_{i}\right)$$

The expression of the sinusoidal signal generated by the receiver is

(6) $$s\left(t\right)=\sin\left(2\pi f_{i}t\right)$$

Therefore, the signals in-phase and quadrature, I(t) and Q(t), are

(7) $$I\left(t\right)=m_{t}\left(t\right)s\left(t\right)=\frac{A_{i}}{2}\left(\cos\phi_{i}+\cos\left(2\pi \left(2f_{i}\right)t+\phi_{i}\right)\right)$$

(8) $$Q\left(t\right)=m_{t}\left(t\right)s\left(t+\pi /2\right)=-\frac{A_{i}}{2}\left(\sin\phi_{i}+\sin\left(2\pi \left(2f_{i}\right)t+\phi_{i}\right)\right)$$

As can be seen, the I(t) and Q(t) signals are composed of two components. A continuous component and another component at twice the frequency. The information to obtain is in the continuous component. By applying a low-pass filter, the information is obtained:

(9) $$I_{F}\left(t\right)=m_{t}\left(t\right)s\left(t\right)=\frac{A_{i}}{2}\left(\cos\phi_{i}\right)$$

(10) $$Q_{F}\left(t\right)=m_{t}\left(t\right)s\left(t+\pi /2\right)=-\frac{A_{i}}{2}\left(\sin\phi_{i}\right)$$

The module $A_{r}$ and phase $\phi_{i}$ of the resulting signal can be obtained with the following expressions:

(11) $$\phi_{i}=\tan^{-1}\frac{I_{F}\left(t\right)}{Q_{F}\left(t\right)}$$

(12) $$A_{r}=\sqrt{I_{F}^{2}\left(t\right)+Q_{F}^{2}\left(t\right)}$$

In this work, it is only necessary to obtain the RMS value of the signal to compute the signal reception point on the PSD surface.

If instead of receiving the signal from only one emitter, the signals of *N* emitters are received, replicas of the signals are produced at the frequencies of $f_{i}-f_{r}$ and $f_{i}+f_{r}$ where $f_{r}$ is the sine wave signal generated in the receiver. To be able to discriminate without interference the signal from each emitter, the low-pass filter used must be less than half the minimum separation between simultaneous frequencies, or in its absence, less than half the smallest separation between a fundamental frequency and one of the harmonics of the other frequencies.

**Figure 6:**
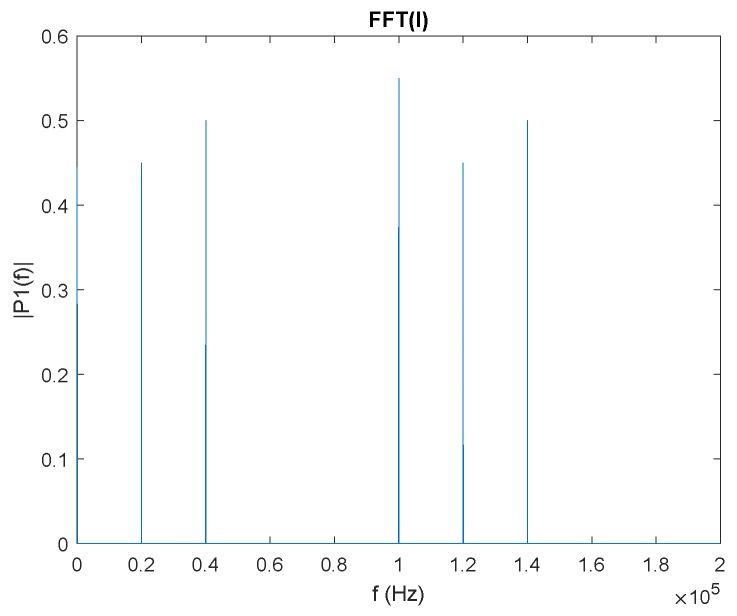
Example of three emitters using FDMA showing the I(t) signal in the frequency domain.

An example with three emitters is shown below. Signal $m_{t}\left(t\right)$ is composed by the sum of the signals of three emitters of frequencies 50, 70, and 90 kHz. Figure 6 shows the FFT of the I(t) signal. It can be seen that there is one component in continuous and another component in 100 kHz, besides the components of the other two emitters at frequencies of (70−50), (70+50), (90−50), and (90+50) kHz.

Once the low pass filter is applied, the RMS value of the emitter working at 50 kHz can be obtained. It should be noted that the RMS value obtained is multiplied by the filter gain factor. Since the same filter is used for all four channels of the PSD, this effect is not relevant.

To implement this system, two multipliers and two low pass filters should be used for each emitter and channel of the PSD. In this case, the digital low pass filter would be the same for all the emitters.

One of the advantages of this technique is that an RMS value per sample of the input signal can be obtained. It is not necessary to wait for an integer number of signal periods to obtain the RMS value.

### 3.2. Code-Division Multiple Access (CDMA)

The CDMA technique is implemented using a binary phase-shift keying (BPSK) modulation because the PSD has a finite bandwidth as well as to drive away, as far as possible, continuous and low-frequency noise. Therefore, the following aspects must be taken into account when deciding on the sequence parameters:

- The chip frequency has to be the same as sinusoidal frequency. This means that each chip modulates an integer period of the sine signal
- The chip frequency and sinusoidal frequencies must be less than or equal to half the sensor’s bandwidth BW. This ensures that the filter accepts frequencies from the main lobe of power spectral density of the signal m(t) which is where most of the signal strength is concentrated.
- The phase of the sine $\phi_{0}$ has to be 0 degrees with respect to the code, so that the symbol transitions happen in the crossing of the sine by 0, reducing the distortion by the BW limitation.

**Figure 7:**
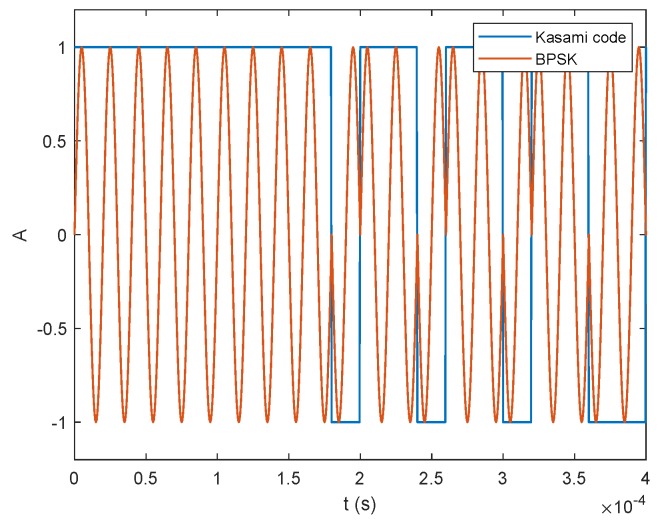
Example of the first chips of a Kasami sequence in conjunction with the modulated signal in BPSK.

Figure 7 shows the first chips of a 1023-chip (10-bit) long Kasami sequence, together with the modulated signal in BPSK. A chip frequency of 50 kHz is used. In this work, we use Pseudo Random (PN) sequences and more specifically Kasami sequences [32] due to the good properties of cross-correlation and autocorrelation, and the number of sequences available, suitable to the number of users in the system.

**Figure 8:**
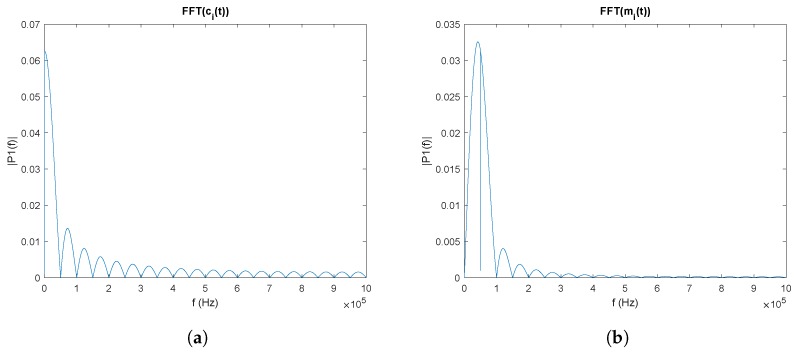
Module of the FFT of: (**a**) the Kasami sequence; and (**b**) of the Kasami sequence modulated in BPSK.

Figure 8a shows the FFT module of a Kasami sequence of 1023 chips. Figure 8b shows the FFT module of the modulated signal in BPSK. It can be seen that the main lobe of c(t) has been shifted to the sine frequency, which is equal to the chip frequency, achieving the greatest amount of information in the available bandwidth and being able to avoid continuous or low-frequency noise.

Once the signal is received in the receiver, a correlation with the same sequence modulated in BPSK would be performed to obtain a correlation peak. This peak is the value that would be obtained to calculate the signal reception point on the PSD surface.

The expression in discrete time to calculate the periodic correlation of two signals $m_{i}\left(t\right)$ and $m_{j}\left(t\right)$ is defined as:

(13) $$R_{m_{i},m_{j}}\left[\tau \right]=\sum_{l=0}^{L-1}m_{i}\left[l\right]m_{j}\left[l+\tau \right]$$

where *L* is the size of the sequence. When i=j, the autocorrelation function is obtained, and, when i≠j, the cross-correlation function is obtained instead.

Applying the correlation properties, the correlation function can be written from the FFT values of the signals according to:

(14) $$R_{m_{i},m_{j}}=\mathrm{IFFT}\left\{\mathrm{FFT}\left\{m_{i}\right\}•\mathrm{conj}\left(\mathrm{FFT}\left\{m_{j}\right\}\right)\right\}$$

where • is a point-to-point multiplication and conj is the conjugated function.

**Figure 9:**
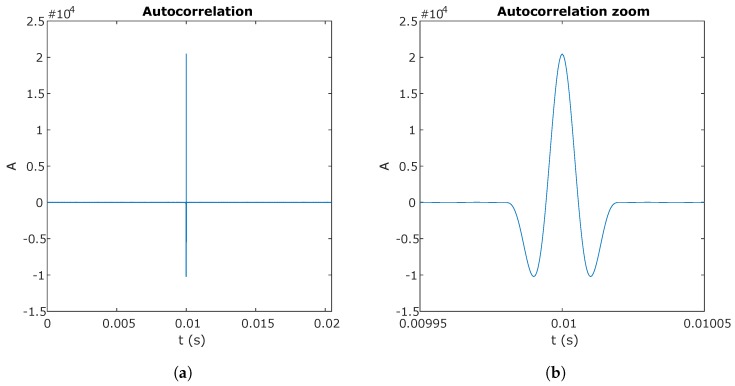
The 10-bit Kasami sequence autocorrelation function modulated in BPSK: (**a**) total size of the autocorrelation function; and (**b**) extended area centered on the correlation peak.

Figure 9 shows the autocorrelation function of a 10-bit Kasami sequence modulated in BPSK, with a chip frequency of 50 kHz and a sampling frequency of 2 Msamples/s. One of the sequences used in the correlation is shifted 10 ms.

One of the main problems of CDMA is Multiple Access Interference (MAI) due to non-zero cross-correlations. There are algorithms to mitigate this effect as much as possible. In our work, we use the cancellation of interference by subtraction explained in [33]. There are two strategies: Successive Interference Cancellation (SIC) and Parallel Interference Cancellation (PIC).

After analyzing the two strategies, it is proposed to implement the option PIC, since it allows a parallel implementation appropriate for hardware systems such as FPGA, and also provides excellent results.

As a summary, the steps for carrying out MAI reduction using PIC are shown below:

- Correlate the received signal $m_{t}\left(t\right)$ with each of the sequences used by the system (different emitters) $m_{i}\left(t\right)$ and obtain the correlation peak ${\hat{A}}_{i}$ and the associated offset ${\hat{\tau }}_{i}$ to each sequence *i*.
- For each *i* sequence, obtain a new ${\hat{m}}_{t_{i}}\left(t\right)$ signal by subtracting from the received signal $m_{t}\left(t\right)$ the contributions received from the rest of the *j* sequences, where j≠i. With this subtraction, the signal from the emitter *i* is filtered from the rest of the sequences. The signal ${\hat{m}}_{j}\left(t\right)$ that is subtracted from the received signal becomes the sequence *j* after passing through the channel and being received by the receiver, that is, the convolution of $m_{j}\left(t\right)$ with the response of the channel h(t). The ${\hat{m}}_{j}\left(t\right)$ signals should be weighted by the peak of the ${\hat{A}}_{j}$ correlation of the received signal $m_{t}\left(t\right)$ with the *j* sequences and divided by the ${\hat{A}}_{j}^{AC}$ autocorrelation of the *j* sequence. This would leave the expression:
  (15) $${\hat{m}}_{t_{i}}\left(t\right)=m_{t}\left(t\right)-\sum_{j=1;j\neq i}^{N}\frac{{\hat{A}}_{j}}{{\hat{A}}_{j}^{AC}}{\hat{m}}_{j}\left(t-{\hat{\tau }}_{j}\right)$$
  where
  (16) $${\hat{m}}_{j}\left(t\right)=m_{j}\left(t\right)⊗h\left(t-{\hat{\tau }}_{j}\right)$$
- Use the ${\hat{m}}_{t_{i}}\left(t\right)$ signal as a received signal and go back to the first step. This means correlate again the sequences, determine the delays, and obtain a subtractive signal. This process could be carried out during several iterations until the amplitude and delay estimates do not vary significantly from one iteration to the next.
- For each ${\hat{m}}_{t_{i}}\left(t\right)$ signal after *q* iterations, the value of ${\hat{A}}_{i}$ and $t\hat{a}u_{i}$ is obtained for each *i* sequence.

In the PSD-based positioning system, this MAI interference produces an offset or bias at the signal reception point on the PSD surface. This offset is due to the differences in RMS value that MAI produces in each of the four channels of the PSD. The offset depends on:

- The delay between sequences. Since there is no synchronism, it is not possible to know beforehand the delay that an emitter will have with respect to the rest of the emitters. The closer the correlation peaks are to each other, the bigger is the interference. Since the correlation peak is not a perfect delta, when measuring the peak value, small interferences between agents produce an offset in the calculation of the signal reception point on the PSD surface.
- The relative signal strength between one emitter and another. If the difference between the signal strength received by one emitter and another is significant, it could be the case that the autocorrelation peak is lower than the cross-correlation, producing errors in the calculation.
- The type of sequences used. Each type of sequence has a different behavior and is more or less affected by the MAI. As already mentioned in this case, Kasami sequences are used.
- Length of the codes. The higher is the number of bits, the higher is the peak of the correlation and the easier it is to detect it. In contrast, more computing time is required to carry out the correlations.
- Emitter positions. The signal reception point on the surface of the PSD depends on the position of each emitter. The level of interference on the impact point for one emitter depends on the relative position of other emitters.
- Number of simultaneous emitters. The greater is the number of simultaneous emitters, the greater is the interference between them. Depending on the length of the codes, a certain number of possible emitters is available. For example, in the case of Kasami codes, the maximum number of emitters depending on the number of bits of the sequence is shown in Table 1.

Therefore, when using codes there are multiple related parameters that make it very difficult to analyze, estimate or correct the offsets produced by the MAI.

## 4. Error Analysis in Emitters’ Position Determination

This section shows the results of simulations and experimental tests analyzing the different parameters that affect the computation of the impact point on the PSD surface and will compare the different multiple-access discrimination techniques.

The influence of the noise is analyzed according to the size of the signal and the technique used. Interference between different emitters is also analysed.

**Figure 10:**
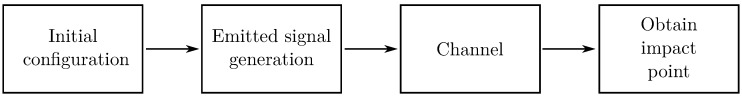
General diagram of the simulator.

As a summary, Figure 10 shows a general scheme of the simulator, composed by the following steps:

- Initial configuration: The general system parameters are configured, such as sampling frequency, type of modulation, number of emitters, and both emitter and receiver characteristics.
- Generation of emitted signal: The signal that is emitted by each of the emitters is generated according to the type of modulation and technique used.
- Channel: This stage simulates the behavior of the channel from the moment the signal is emitted by the emitters until it is finally received by the PSD sensor.
- Obtaining the impact point: In this stage, the impact point is obtained from the signal of each of the the Table channels of the PSD. Depending on the modulation used, the RMS value is obtained differently.

Depending on the parameter to be analyzed, one configuration or another is chosen. In each of the next sections, both the set-up and the tests carried out are detailed.

**Figure 11:**
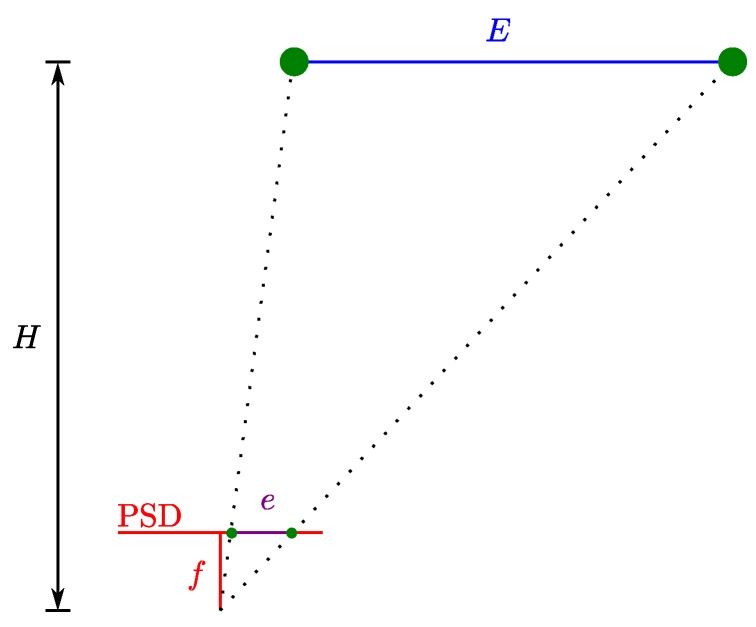
Diagram showing the correspondence between errors on the surface of the PSD and positioning errors.

It should be noted that the shown errors (*e*) are the impact point errors in the PSD surface. To calculate positioning errors (*E*), the impact point errors must be multiplied by a factor of H/f:

(17) $$E=\frac{H}{f}e$$

where *H* is the height of the environment and *f* is the focal length. Figure 11 shows a diagram that represents the correspondence between errors on the surface of the PSD and positioning errors.

### 4.1. Behavior of the Different Techniques in Noisy Environments

In this section, our aim is to observe the behavior of each technique in a noisy environment. To isolate the rest of the effects, a single emitter and a single receiver are used. Environments with different noise levels are simulated and errors in the calculation of the impact point are compared using different signal lengths with the following techniques: FDMA with filters, FDMA with FFT, FDMA with IQ, and CDMA modulated in BPSK.

#### 4.1.1. Test Scenario

The emitter is located in the z=3 m plane, at the coordinates (0,0,3) m, with its surface vector perpendicular to the z=3 m plane. The emitter is modulated with a frequency of 50 kHz. Therefore, when analyzing FDMA, the emitter outputs a 50 kHz tone, and, when analyzing CDMA, the emitter outputs a 50 kHz chip frequency sequence modulated in BPSK with a 50 kHz sine signal.

**Figure 12:**
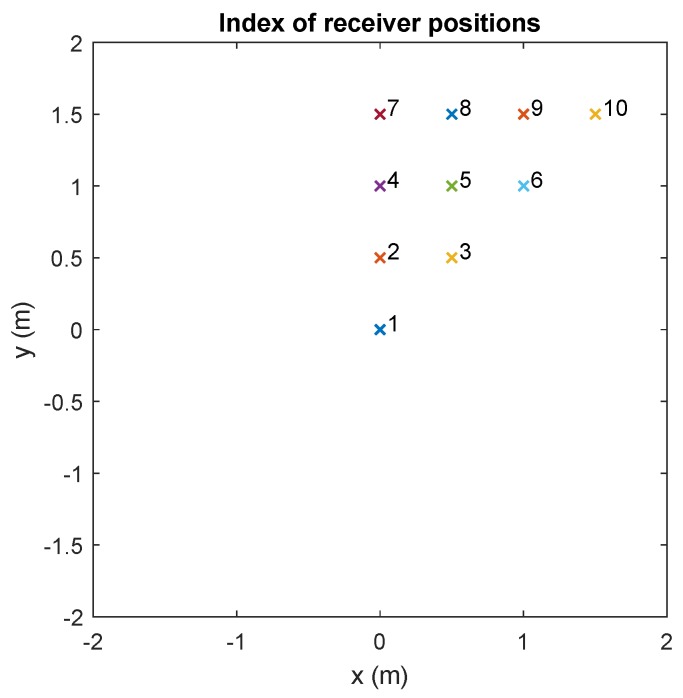
Indexes of the different receiver positions.

The receiver moves to different points on the z=0 m plane maintaining the same orientation in all tests. The receiver moves to positions on the z=0 m plane shown in Figure 12. These 10 positions were chosen because, with them, by means of symmetry, the effects can be observed on practically the entire surface of the sensor. The receiver receives the data with a sampling frequency of $f_{s}=1$ Msample/s.

In total, 11 noise scenarios were simulated with an SNR between 57 and 107 dBHz in 5 dBHz steps, considering a bandwidth of $f_{s}/2$ Hz. Appendix A describes how the noise signal was generated. For example, considering a bandwidth $f_{s}/2$, in the cases of 57 and 107 dBHz, SNR in dB calculated as SNR=10log10(Ps/Pn), is 0 and 50 dB, respectively.

Two different signal lengths were simulated, corresponding to 8- and 10-bit Kasami sequences. Therefore, a new position of the point of impact was calculated each time:

(18) $$N^{\circ }\mathrm{samples}=\left(2^{L}-1\right)S_{c}$$

where *L* is the number of bits in the sequence and $S_{c}$ the samples per chip, which in this case is 1M/50k=20 samples per chip.

In the case of FDMA, it would not be necessary to use so many samples to obtain the RMS value, but, since we wanted to compare these techniques, we used the same number of samples.

#### 4.1.2. Results

For each of the techniques, Table 2 and Table A1, Table A2, Table A3, Table A4, Table A5, Table A6 and Table A7 show the average, maximum, and standard deviation values for each of the receiver position indices as well as for each of the SNRs analyzed. The results in general terms can be said to be very similar for all three techniques and it can be seen that the error is approximately half when using 1023 signal periods (10 bits) than when using 255 periods (8 bits).

**Figure 13:**
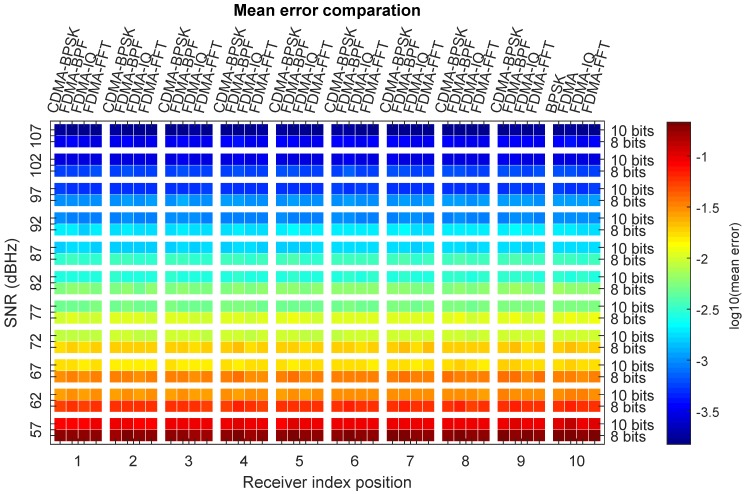
Comparison of the average error in the calculation of the impact point as a function of the SNR, the position of the receiver, technique used, and signal size.

**Figure A1:**
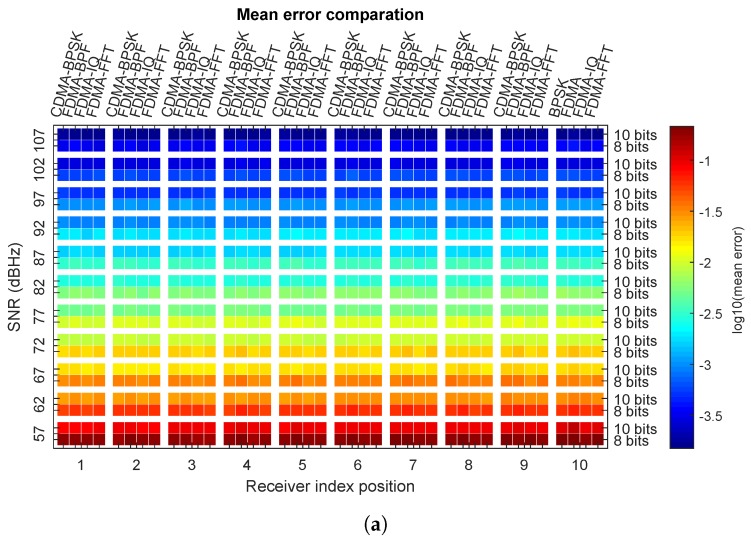

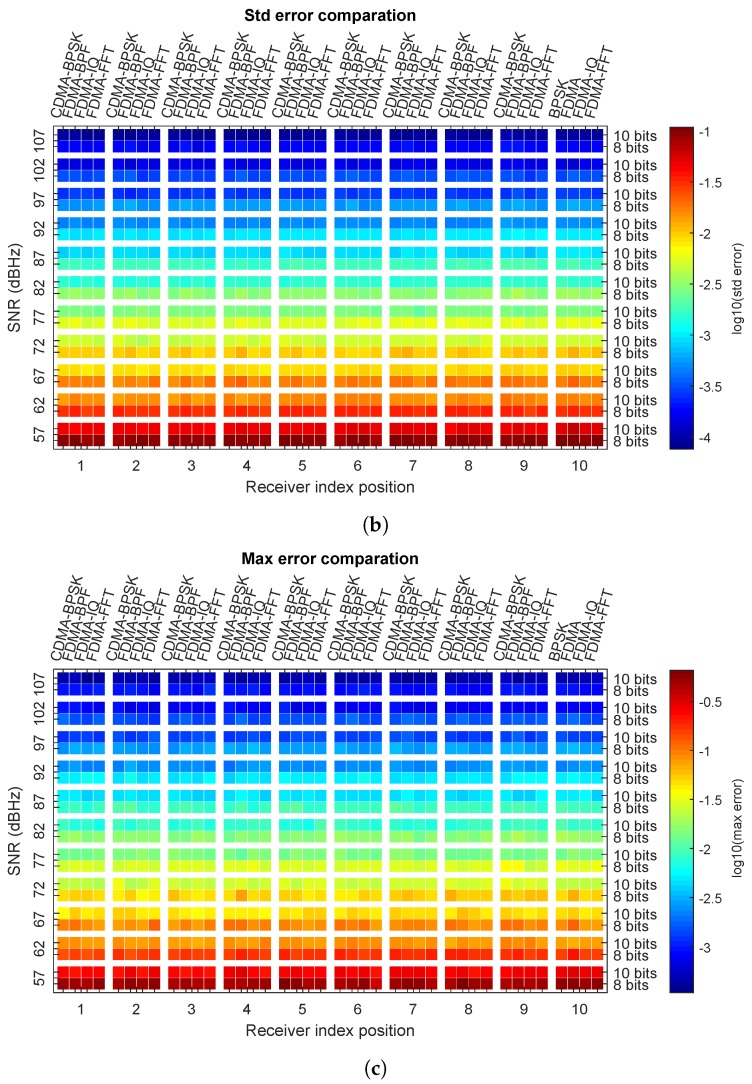
Comparison of the error in the calculation of the impact point as a function of the SNR, position of the receiver, technique used, and signal size: (**a**) mean error; (**b**) standard deviation error; and (**c**) maximum error.

To observe more visually the results obtained, a comparison is shown of the mean value, standard deviation, and maximum error value in Figure 13 and Figure A1b,c, respectively. The figures show for each receiver position index and for each SNR value the errors for each of the three techniques (CDMA-BPSK, FDMA, FDMA-IQ, and FDMA-FFT) in the two signal sizes used (8 and 10 bits). The value of the error is represented by a different color. To better observe the differences, the value of log10(error) is shown.

From the values observed in the tables and figures, two important conclusions can be drawn. Firstly, it can be seen that the error decreases as the size/length of the signal increases. This is logical because, as it is Gaussian noise with a zero average, by averaging more samples, its effect is reduced. The second conclusion that can be drawn is that the differences among the four types of techniques are not very significant. Therefore, it can be said that the four techniques behave very similarly in noisy scenarios with only one emitter. Therefore, the noise is not an influential parameter for deciding on one technique or another.

**Figure 14:**
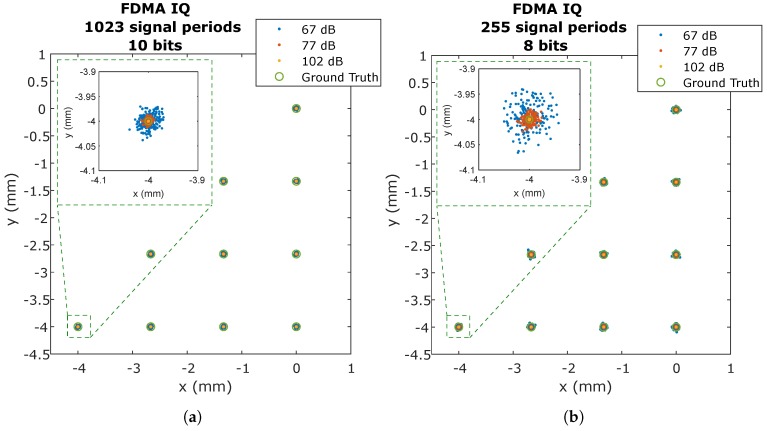
Impact points on the PSD for each of the positions where the receiver is positioned according to the SNR using FDMA with IQ and size of: (**a**) 10 bits; and (**b**) 8 bits.

To see more visually the effect of noise in determining the impact point, Figure 14a,b show, as an example, the case of FDMA with IQ for the equivalent signal sizes of 8 and 10 bits, for the signal-to-noise ratios of 67, 77, and 102 dBHz.

In Figure 14a,b, it can be seen how the point cloud gets smaller as the SNR increases. If we compare Figure 14a,b, we can see, as commented above, that the dispersion of the points is more significant if the length of the signal to be processed is shorter. The rest of the techniques are similar. It should be noted that, in a real environment, with the emitter located at 3 m, the SNR of the signal is around 77–82 dBHz, depending on the emitted signal strength, lens, etc.

### 4.2. Interference Analysis of the Different System Emitters

In this section, we analyze how each of the techniques works while obtaining the point of impact of several emitters emitting simultaneously.

After analyzing the FDMA technique, it can be said that as long as the number of emitters is not excessively high (in this system, it is not intended that more than four emitters be emitted simultaneously), there is no interference between the different emitters. This is because the emitters being used are emitting a very clean signal. This, together with the narrow filters that have been implemented means that there is practically no interference between emitters. The possible interference that could appear would be due to harmonics; thus, if the frequencies used are chosen correctly, this effect should not have any influence.

As for the CDMA technique, it has been observed that it does present interference due to MAI. As mentioned above, there are several variables that influence MAI interference, such as the number of simultaneous emitters, their positions, the position of the receiver, gaps between the different emitters, etc. Therefore, this section shows some examples of the effect of MAI in different scenarios and how the PIC algorithm manages to reduce its effects.

#### 4.2.1. Test Scenario

Since there is an infinite number of possible parameter combinations, a configuration with three emitters is considered as an example.

**Figure 15:**
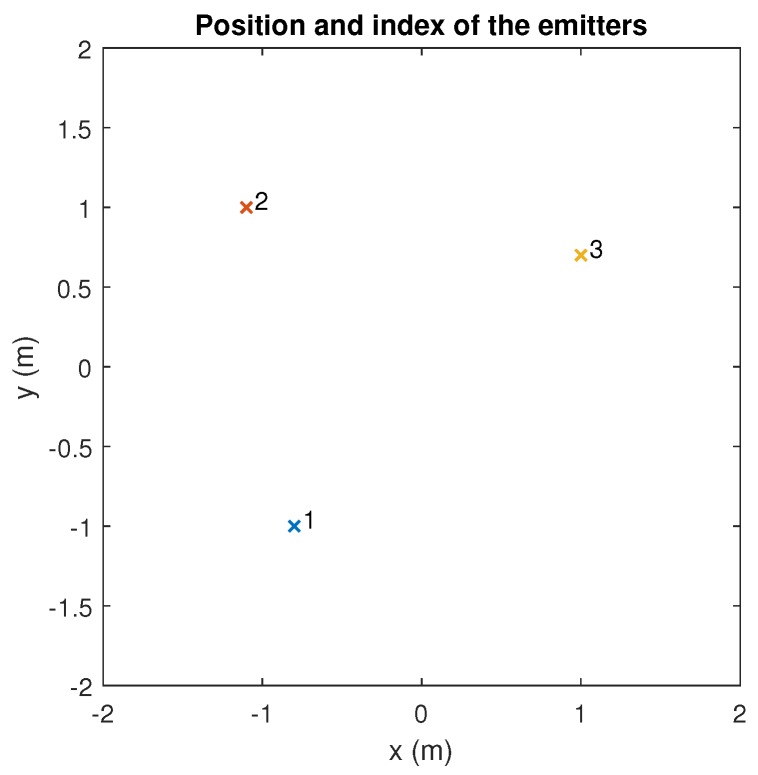
Position and index of the different emitters.

The emitters were placed on the plane of the ceiling at the height of 3 m above the floor, oriented vertically downwards, at the points shown in Figure 15. The receiver was placed on the floor, co-planar with the plane of the floor, at the coordinates (0,0,0) m.

#### 4.2.2. Results

**Figure 16:**
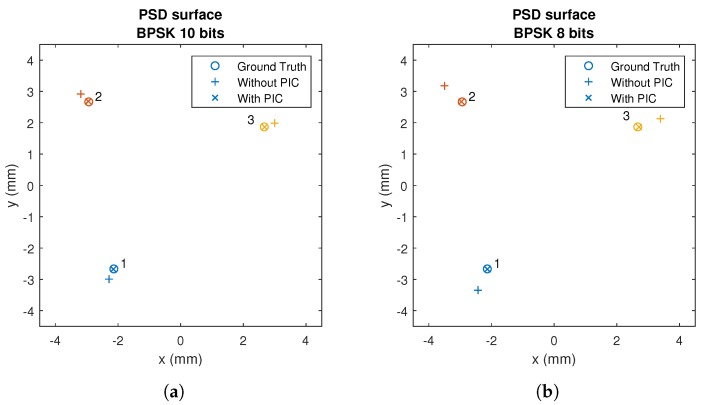
Impact points on the PSD of the five emitters using CDMA-BPSK before and after applying the PIC correction with sizes of: (**a**) 10 bits; and (**b**) 8 bits.

Figure 16 shows the impact points on the surface of the PSD, for a sequence length of 8 and 10 bits. The points without applying PIC are shown with crosses and the points applying PIC are shown with blades.

Figure 16 shows the effect of the MAI on the detection of the point of impact. The MAI causes a shift from the actual point. To reduce this effect, the PIC algorithm was implemented.

In Figure 16, it can be seen how the PIC algorithm significantly reduces the displacement of the point of impact due to MAI. It can also be seen clearly how this offset depends on the size of the signal and, therefore, on the code used.

**Figure 17:**
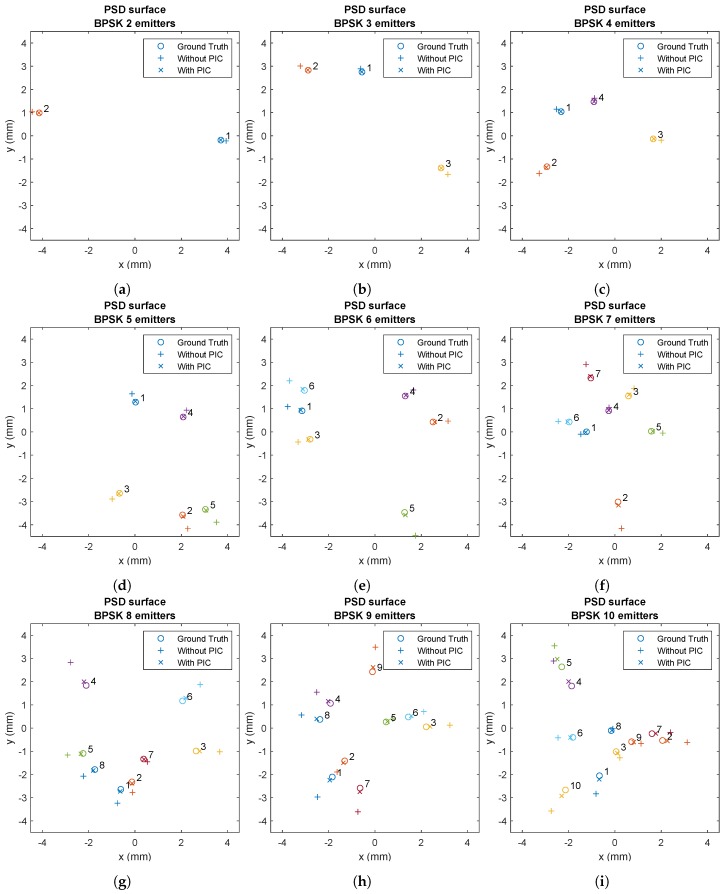
Impact points in the PSD using CDMA-BPSK with 10 bit size before and after applying correction with: (**a**) two emitters; (**b**) three emitters; (**c**) four emitters; (**d**) five emitters; (**e**) six emitters; (**f**) seven emitters; (**g**) eight emitters; (**h**) nine emitters; and (**i**) ten emitters.

Figure 17 shows several examples where the effect of MAI and its correction with the PIC algorithm can be seen visually. Nine different tests were carried out in which the number of simultaneous emitters was varied from 2 to 10. The emitters were randomly positioned within the FoV of the receiver and a zero offset was set between the transmission of each of the sequences. In all cases, 10-bit Kasami code was used.

It can be seen in all cases how this displacement or offset is greatly reduced in the determination of the impact point. However, it should be noted that the correction is not total. This is mainly due to the following causes:

- Only one iteration of the algorithm was used. It was found that increasing the number of iterations improves the correction very little, and, conversely, greatly increases complexity, resources, and execution time.
- It is very difficult to model correctly the signal that is used to perform the subtractions. Ideally, it should be the convoluted sequence with the channel. In this case, the channel was modeled experimentally, but there are always small differences or errors. In the simulation, a small noise was added to model this lack of accuracy in modeling.
- In practical examples, the time lag with which the sequence arrives at the PSD will hardly be an integer number of samples. This is because, to simplify the implementation of the receiving system, the correlation is made and the peak and peak position are obtained. That position will be an integer number of samples. Therefore, there is a resolution of $1/f_{s}$ s. This situation means that, when performing the subtraction, the correct signal is not exactly subtracted, since in the simulator when moving the signals if non-integer samples have been taken into account. To reduce this effect as much as possible, there are techniques such as Early–Late [34], in which two correlations are made, one forward and one backward in a given delta. Using Early–Late, the signal offset accurately measured below the sample can be obtained. That algorithm was implemented getting the lag quite close to the correct one, but it presents a problem with its execution time, complexity, and resource consumption. It should be noted that once, the time lag below the sample has been obtained, it needs to be applied to the signal. To achieve the desired offset, the signal would have to be oversampled and interpolated at a higher sampling rate than $f_{s}$, an integer number of samples would have to be shifted, and, finally, downsampling would have to be performed. After the tests, it was decided not to implement it in the final system, since the improvement it provided was not comparable with the complexity required to carry it out.
- This simulation was carried out in the absence of noise. If noise were added (receiver, emitter, or channel noise), it would be more challenging to obtain the remaining signal with which to properly correct.
- As the number of emitters increases, there is more interference due to MAI, and, therefore, it is more complicated to correct.

Let us summarize the different configuration settings after performing the synthetic analysis using one single emitter. The results obtained, in terms of position determination error, are similar in all the analyzed configurations and are a function of the available SNR.

The error results integrating 1023 signal cycles (or sequences of 1023 symbols in the case of CDMA) reduces at the half the error of 255 signal cycles case.

When working simultaneously with multiple emitters (multiple-access to the channel), it is concluded that any of the techniques used in FDMA present interferences between signals that are not significant for the positioning determination; however, working with CDMA, the MAI interferences are significant and cannot be assumed in these systems.

In the case of CDMA multiple-access, a correction must be used based on the PIC algorithm, which is capable of mitigating interference errors as long as the number of different sequences does not exceed four. If this number is exceeded, the errors must be taken into account. Besides, it should be considered that the PIC algorithm requires some execution time that may not allow the system to work in real-time.

## 5. Experimental Tests in Real Environments

In this section, different tests performed in a real environment are shown to decide which technique is more suitable for use in the LPS system.

Firstly, we analyze the interference results using FDMA and CDMA. Then, we analyze the error in the determination of impact points using FDMA, under different SNR and integration time conditions.

**Figure 18:**
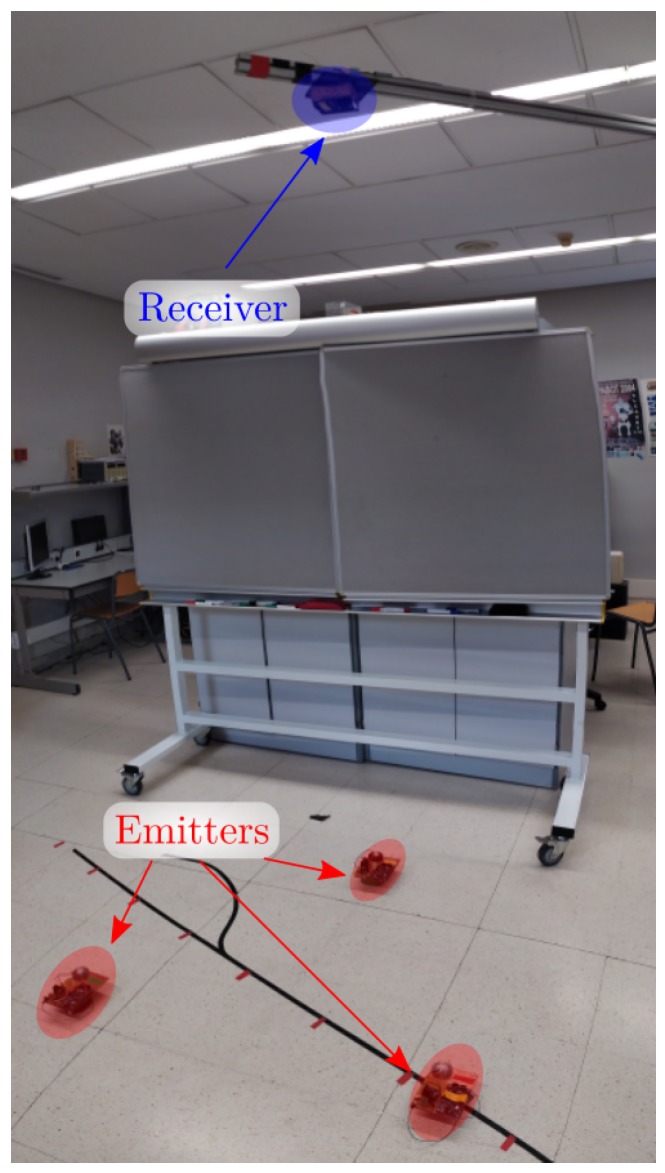
Set-up of the experimental tests.

**Figure 19:**
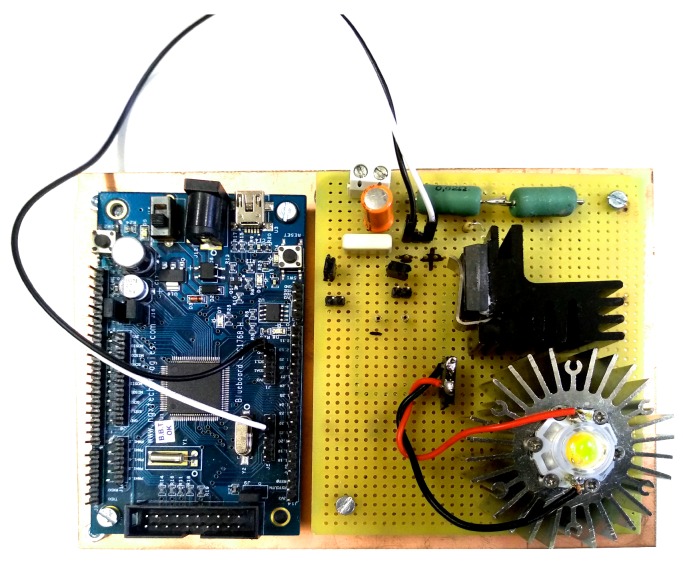
One of the emitters used in the experimental tests.

**Figure 20:**
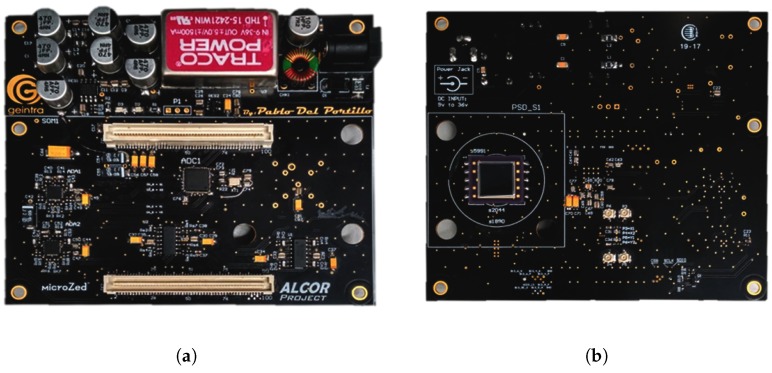
The receiver used in the experimental tests, (**a**) back and (**b**) front.

The set-up of the experimental test is shown in Figure 18, one of the emitters used in the experimental tests is shown in Figure 19, and the receiver is shown in Figure 20.

### 5.1. Interference Results Using FDMA and CDMA

Three emitters were placed on the same plane 3 m away from the PSD sensor, 90 cm apart. A 9 mm × 9 mm PSD sensor with an 8 mm focal length lens was used. The acquisition was made at 1 MHz.

The techniques of CDMA-BPSK and FDMA-IQ were analyzed. In the case of CDMA- FDMA, a chip frequency of 5 kHz and 8- and 10-bit Kasami sequences (255 and 1023 chips, respectively) were used. In the case of FDMA, each emitter emitted at 6, 8, and 10 kHz.

**Figure 21:**
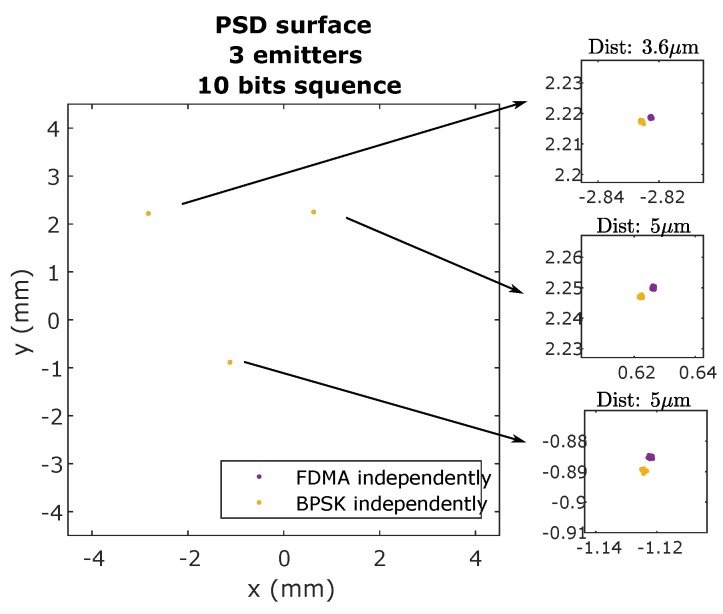
Impact points on the PSD surface using FDMA and using independently emitted BPSK sequences.

Firstly, for both CDMA-BPSK and FDMA-IQ, the signal from each emitter was acquired separately, to avoid possible interference between emitters. The impact points obtained in the PSD, for the 10-bit case, are shown in Figure 21.

It can be seen that practically the same points of impact were obtained with both techniques, with differences in the surface of the PSD of around 5 μm. In this scenario, the SNR was around 66 dB.

**Figure 22:**
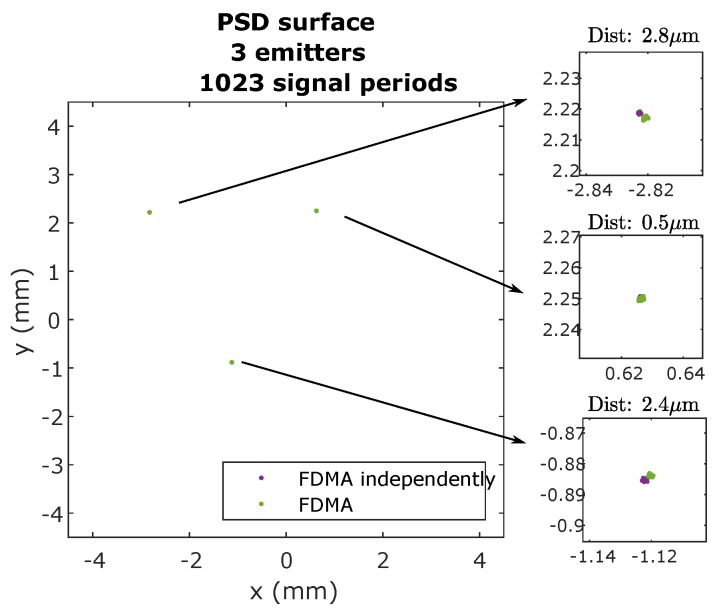
Impact points on the PSD surface using FDMA and using independently emitted BPSK sequences.

Figure 22 shows the points of impact using the FDMA technique, when all three emitters emit simultaneously and when they emit independently. In this case, the interference can be assumed to be practically zero.

**Figure 23:**
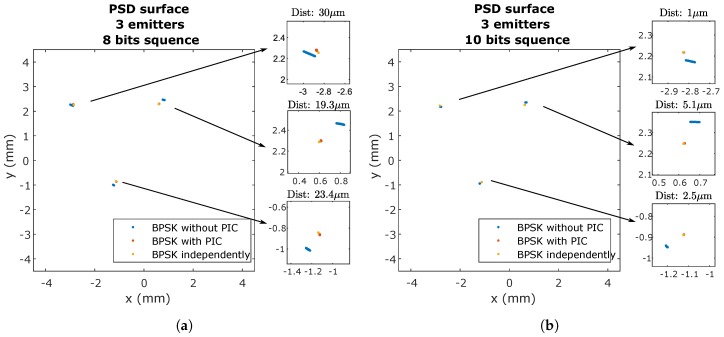
Impact points on the PSD surface using CDMA-BPSK emitted independently, not applied and applying PIC correction with a size of: (**a**) 8 bits; and (**b**) 10 bits.

The same procedure was done for CDMA-BPSK, and the results are shown in Figure 23 for 8- and 10-bit sequences. The distance shown in Figure 23 is the distance between BPSK, emitting the three emitters simultaneously and correcting the interferences with PIC algorithm respect to BPSK emitting the three signals independently (no simultaneously).

It can be observed whether or not there is interference between emitters. Before making the correction, there were different offsets for the same emitter. This is due to the differences between the emission and sampling frequencies that are set and the real frequencies, as well as the jitter of the clocks. These differences cause minimal signal mismatches that alter the obtaining of the correlation peak and modify the MAI interference. Once PIC was applied, the effects were significantly corrected. The larger is the signal size, the better is the correction and the less it is affected by noise.

After analyzing the results, it can be said that, using the FDMA technique, there are no interferences between emitters. It was also seen that applying the PIC algorithm is able to greatly reduce MAI interference in CDMA, but it is necessary to implement the algorithm by increasing the complexity of processing in the receiver.

Therefore, after analyzing the results described thus far, it can be said that the best option in our particular system is the implementation through FDMA, since the interference between the different emitters is practically zero. Among the three options, the IQ demodulator option was chosen, since it allows obtaining the RMS sample by sample without having to wait for an integer number of periods of the signal.

### 5.2. Error in the Determination of Impact Points Using FDMA, under Different SNR and Integration Time Conditions

In this section, we analyze the FDMA-IQ technique in a noisy scenario considering different integration times in the receiver. With this, we intend to estimate which errors in the estimation of the reception point of the signals on the surface of the PSD depend on the rate of desired measurements per second and the SNR of the environment.

**Figure 24:**
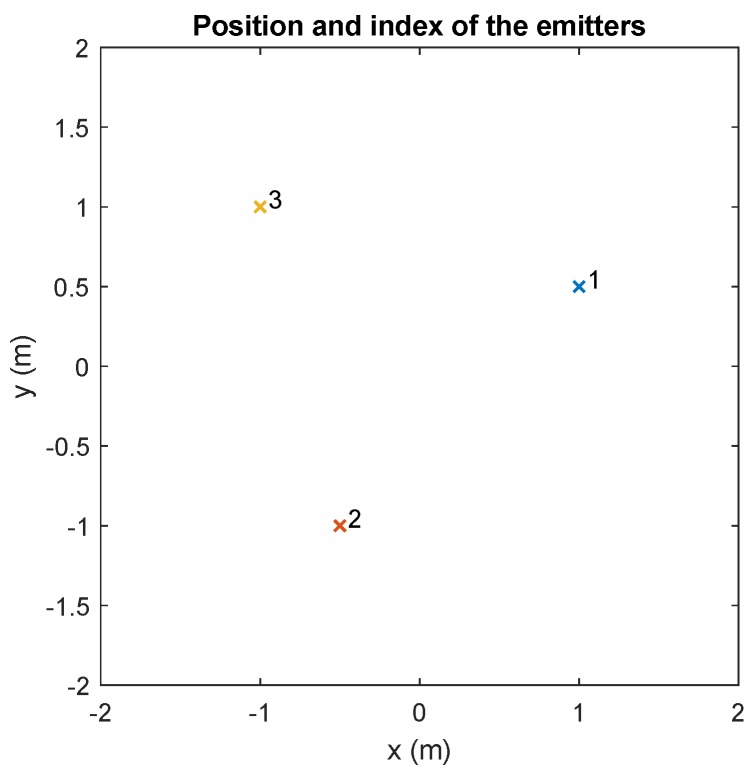
Position and index of different emitters.

The simulations were carried out in a scenario with three emitters located in the positions shown in Figure 24, in the plane of the ceiling, 3 m from the floor, and oriented vertically downwards. Each emitter sent a sinusoidal signal at 50, 70, and 90 kHz (different frequency each one). The receiver was located at the coordinates (0,0,0). A sampling frequency of 1 Msamples/s was used.

The simulations were performed by varying the SNR from 23 to 73 dB in 5 dB steps. We used band pass filters with 1 kHz bandwidth. For each SNR value, the signal reception point on the PSD surface was obtained depending on the signal integration time. Knowing the sampling frequency and the integration time of the processed signal, it is possible to obtain the range of data per second that we can obtain. Table 3 shows the different values of the signal integration time (or the number of periods/samples) used, as well as the range of position measurements per second.

**Figure 25:**
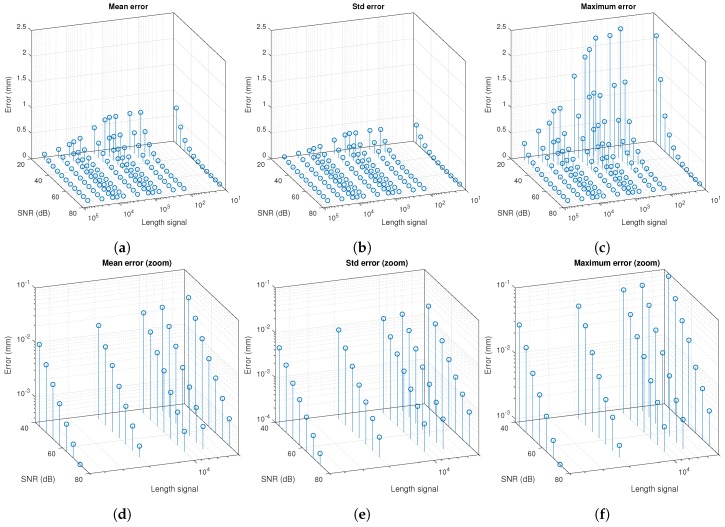
Error at the reception point on the PSD surface of each emitter as a function of signal size and RMS value: (**a**) mean value; (**b**) standard deviation; and (**c**) maximum value. Extended error area between 5000 and 50,000 samples and between 43 and 73 dB with the error axis in logarithmic scale: (**d**) mean value; (**e**) standard deviation; and (**f**) maximum value.

From the simulation, Figure 25 shows the value of the mean, standard deviation, and maximum error in the determination of the reception point on the PSD surface, for each of the three emitters. The error is measured as the Euclidean distance between the actual point and the obtained point. The values are shown as a function of the SNR and the signal integration time. Table 4 shows the results of the simulation.

**Figure 26:**
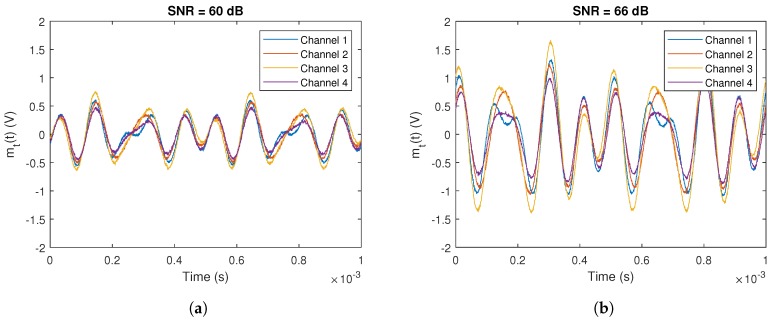
First samples of the measured signal of the empirical tests with SNR of: (**a**) 60 dB; and (**b**) 66 dB.

Table 5 shows the results of experimental tests. It shows the error in function of the signal length (in samples) and SNR. To carry out the empirical tests, we considered the same setup used in previous section with three emitters using FDMA. We implemented the IQ demodulation in the receiver to be able to compare with the simulations results. To analyze two different SNR scenarios, the three emitters were configured with different signal strengths to obtain a SNR in the receiver of 60 and 66 dB. Figure 26 shows the first samples of the empirical tests in both SNR scenarios.

The simulation results are given in Table 4. These data show that simulation and empirical results have very similar values, validating the simulation procedure.

Figure 25 shows how the errors become smaller as the SNR increases. The same happens when the integration time to process is increased. These figures allow knowing the error according to the SNR and the signal integration time. Therefore, if the SNR of the received signal is known or can be estimated a priori, with the information in Figure 25, the number of samples (integration time) that must be used to be below a certain error can be established. In the same way, if a certain range of measurements per second is needed, inversely proportional to the size of the signal, it is possible to know what SNR the received signal must have to be below a certain error. For example, to obtain that certain SNR, the emission signal strength could be increased.

## 6. Conclusions

This paper analyzes LPS systems with different techniques of discrimination of signals received in a detector, coming from different emitters. The advantages and disadvantages of each of them have been discussed.

FDMA (with three possible implementations: band-pass filters, FFT, and IQ demodulation) and CDMA techniques have been analyzed. The use of TDMA has been ruled out because it needs synchronism between emitters and receiver.

Several simulations were carried out in which the error in the determination of the reception point of the signals on the surface of the PSD was compared (previous step to determine the AoA). First, it was possible to verify that, using only one emitter in a noisy environment, all the techniques analyzed performed correctly, with similar results. It was found that, as the signal capture time increased, or when the number of chips in sequences increased, the error decreased because the noise was averaged.

It was studied how the system determines the different signals from each emitter, proving that with FDMA there is no interference as long as the width of the band pass filters does not overlap in adjacent filters and the number of emitters is not excessively high (in this system, it is not intended that more than four emitters be emitted simultaneously). The scalability of the system is maintained as long as the selection of frequency in a larger area is correctly distributed to not capture emitted signals with the same frequency. Even with only two emitters, we can perfectly obtain the position in planar motion. Additionally, the current BW of the system allows increasing more frequency values.

However, when CDMA is used there is MAI interference between the signals of the different emitters. This interference causes an offset in the determination of the reception point of the signals on the PSD surface. MAI interference depends on many parameters such as type of sequence used, length, phase shift, emitted signal strength, etc. To compensate for the effects of MAI, the PIC algorithm has been implemented achieving a large reduction in the offset produced by the interference. Simulations and experimental tests show the effect of the MAI and how the PIC algorithm succeeds in reducing its effects.

Since the interferences between emitters are practically nonexistent in the FDMA technique (without applying any additional processing), and considering that the PIC algorithm is recursive and needs a relatively long time to be executed (although it manages to eliminate the interferences between sequences), the technique that seems to be more appropriate to be used is FDMA in the development of our LPS.

The use of a particular FDMA implementation in the receiver system depends on its hardware. If FPGA/VLSI is used, the best implementation is IQ demodulation because allows to obtain a signal module value from each input sample. Additionally, the hardware implementation can include a digital filter. On the other hand, if a microcontroller is used, the best option is the FFT implementation because microcontrollers work in sequential mode compared with digital filtering, which requires high computer load and long time. However, if FFT implementation is used, the microcontroller program only must call the FFT function four times, one for each channel of the PSD. Another option with the microcontroller is to use the Goertzel algorithm to obtain the magnitude of signal frequencies, which is the fastest option.

Using FDMA, simulations in a realistic environment and empirical tests were performed. In these tests, the PSD received the signals of three emitters in different SNR conditions. For each SNR, the impact point was obtained as a function of the signal integration time. Obtained results allow knowing which integration time would be necessary to use according to the SNR of the received signal.

The results indicate that it is possible, under normal working conditions, to make more than 50 AoA determinations of different signals per second. In this case with a SNR of 60 dB, the error in the impact point is 3 μm, therefore the positioning error obtained using Equation (17) is 1.1 mm.

## Figures and Tables

**Table 1:** Length of sequences and maximum number of emitters in Kasami sequences.

Number of Bits	Length	Maximum Number of Emitters
N	$2^{N}-1$	
4	15	4
6	63	8
8	255	16
10	1023	32
12	4097	64

**Table 2:** Mean values, standard deviation, and maximum error values in the calculation of the impact point using FDMA with IQ with a 10-bit size.

Index	Error (mm)	57 dBHz	62 dBHz	67 dBHz	72 dBHz	77 dBHz	82 dBHz	87 dBHz	92 dBHz	97 dBHz	102 dBHz	107 dBHz
1	Mean	0.085	0.027	0.015	0.0087	0.005	0.0028	0.0017	0.00089	0.00049	0.00026	0.00016
1	Std	0.048	0.016	0.0079	0.0046	0.0026	0.0015	0.00079	0.00045	0.0003	0.00015	$7.6\times 10^{-5}$
1	Max	0.23	0.076	0.039	0.023	0.013	0.0076	0.004	0.0021	0.0014	0.00079	0.00034
2	Mean	0.083	0.027	0.014	0.0083	0.005	0.0028	0.0015	0.0009	0.00049	0.00028	0.00016
2	Std	0.043	0.015	0.0077	0.004	0.003	0.0015	0.00083	0.00046	0.00026	0.00016	$8.5\times 10^{-5}$
2	Max	0.2	0.074	0.041	0.022	0.015	0.009	0.0044	0.0023	0.0013	0.0008	0.00044
3	Mean	0.09	0.026	0.016	0.0093	0.0049	0.0027	0.0016	0.00094	0.0005	0.00028	0.00018
3	Std	0.048	0.013	0.008	0.005	0.0027	0.0015	0.00078	0.00047	0.00027	0.00015	$8.3\times 10^{-5}$
3	Max	0.23	0.062	0.038	0.025	0.014	0.0092	0.0038	0.0024	0.0015	0.00074	0.0005
4	Mean	0.087	0.029	0.016	0.0084	0.0051	0.0029	0.0016	0.00089	0.0005	0.00028	0.00016
4	Std	0.047	0.014	0.0081	0.0044	0.0026	0.0015	0.0009	0.00049	0.00028	0.00014	$8.4\times 10^{-5}$
4	Max	0.25	0.077	0.041	0.023	0.016	0.0085	0.0049	0.0027	0.0016	0.00075	0.00044
5	Mean	0.092	0.027	0.017	0.0089	0.005	0.0028	0.0015	0.00086	0.0005	0.00028	0.00016
5	Std	0.047	0.014	0.0087	0.0046	0.0028	0.0015	0.00078	0.00044	0.00025	0.00015	$8.6\times 10^{-5}$
5	Max	0.25	0.071	0.05	0.027	0.014	0.0065	0.0049	0.002	0.0015	0.00078	0.00049
6	Mean	0.094	0.03	0.017	0.0092	0.0049	0.0027	0.0017	0.00092	0.00054	0.0003	0.00016
6	Std	0.051	0.016	0.0093	0.0049	0.0026	0.0015	0.00089	0.00048	0.00027	0.00016	$8.1\times 10^{-5}$
6	Max	0.26	0.078	0.045	0.025	0.014	0.0082	0.0044	0.0025	0.0013	0.00079	0.00041
7	Mean	0.092	0.028	0.014	0.0099	0.005	0.0028	0.0015	0.00093	0.00052	0.00029	0.00016
7	Std	0.047	0.016	0.008	0.0048	0.0023	0.0016	0.00085	0.00047	0.00026	0.00015	$8.6\times 10^{-5}$
7	Max	0.28	0.075	0.046	0.025	0.012	0.008	0.0042	0.0022	0.0013	0.00069	0.00041
8	Mean	0.089	0.029	0.016	0.0087	0.005	0.0028	0.0016	0.00088	0.0005	0.00028	0.00016
8	Std	0.05	0.015	0.0088	0.0042	0.0026	0.0015	0.00079	0.00045	0.00026	0.00015	$8.2\times 10^{-5}$
8	Max	0.22	0.071	0.051	0.024	0.013	0.0089	0.0044	0.0024	0.0014	0.00077	0.00044
9	Mean	0.093	0.031	0.016	0.0092	0.0051	0.0029	0.0016	0.00093	0.00049	0.00028	0.00016
9	Std	0.047	0.017	0.0084	0.005	0.0025	0.0015	0.00073	0.00048	0.00026	0.00015	$9.1\times 10^{-5}$
9	Max	0.28	0.092	0.043	0.028	0.012	0.0093	0.004	0.0023	0.0012	0.00076	0.00044
10	Mean	0.095	0.033	0.017	0.0095	0.0053	0.003	0.0017	0.00099	0.0005	0.00029	0.00017
10	Std	0.054	0.015	0.0085	0.0046	0.0029	0.0015	0.00098	0.00048	0.00027	0.00015	$8.8\times 10^{-5}$
10	Max	0.26	0.076	0.042	0.022	0.014	0.0084	0.0056	0.0026	0.0013	0.00082	0.00049

**Table 3:** Signal integration time and range of position measurements per second used in the simulation.

**Length (Samples)**	10	100	200	500	750	1000	2000	5000	7500	10,000	20,000	50,000
**Range (Position Measurements/s)**	100,000	10,000	5000	2000	1333.33	1000	500	200	133.33	100	50	20

**Table 4:** Mean values, standard deviation, and maximum values of the mean error of the three emitters in the calculation of the reception point on the PSD surface using FDMA with IQ as a function of SNR and signal size.

Signal Length (Samples)	Error (mm)	23 dB	28 dB	33 dB	38 dB	43 dB	48 dB	53 dB	58 dB	63 dB	68 dB	73 dB
10	Mean	0.69	0.4	0.22	0.13	0.07	0.04	0.023	0.013	0.0071	0.004	0.0023
10	Std	0.36	0.21	0.12	0.067	0.037	0.021	0.012	0.0067	0.0037	0.0021	0.0012
10	Max	2.1	1.3	0.69	0.4	0.23	0.13	0.072	0.045	0.023	0.014	0.0069
100	Mean	0.69	0.4	0.22	0.13	0.069	0.04	0.022	0.013	0.0072	0.004	0.0022
100	Std	0.36	0.21	0.12	0.065	0.037	0.02	0.012	0.0066	0.0038	0.0021	0.0012
100	Max	2.3	1.4	0.67	0.39	0.25	0.12	0.074	0.04	0.025	0.012	0.0073
200	Mean	0.7	0.4	0.23	0.12	0.07	0.04	0.022	0.012	0.0071	0.0041	0.0022
200	Std	0.37	0.21	0.12	0.064	0.037	0.021	0.012	0.0065	0.0037	0.0021	0.0012
200	Max	2.2	1.3	0.72	0.39	0.25	0.13	0.071	0.04	0.022	0.013	0.0072
500	Mean	0.68	0.39	0.22	0.12	0.069	0.039	0.022	0.012	0.007	0.0039	0.0022
500	Std	0.35	0.2	0.12	0.064	0.036	0.02	0.012	0.0065	0.0036	0.0021	0.0012
500	Max	2.2	1.2	0.73	0.37	0.21	0.11	0.07	0.038	0.023	0.013	0.0066
750	Mean	0.67	0.38	0.21	0.12	0.068	0.038	0.021	0.012	0.0069	0.0037	0.0022
750	Std	0.36	0.2	0.11	0.062	0.036	0.02	0.011	0.0064	0.0036	0.0019	0.0011
750	Max	2	1.2	0.67	0.39	0.21	0.11	0.068	0.04	0.022	0.012	0.0068
1000	Mean	0.63	0.36	0.2	0.12	0.065	0.037	0.021	0.011	0.0065	0.0037	0.0021
1000	Std	0.34	0.19	0.11	0.06	0.033	0.02	0.011	0.0059	0.0033	0.0019	0.0011
1000	Max	1.9	1.2	0.61	0.35	0.19	0.12	0.066	0.034	0.018	0.012	0.0069
2000	Mean	0.51	0.29	0.16	0.092	0.052	0.029	0.016	0.009	0.0052	0.0029	0.0017
2000	Std	0.27	0.15	0.085	0.047	0.026	0.015	0.0086	0.005	0.0027	0.0015	0.00088
2000	Max	1.5	0.81	0.45	0.27	0.16	0.086	0.048	0.029	0.015	0.0089	0.0052
5000	Mean	0.34	0.19	0.11	0.06	0.036	0.02	0.011	0.0061	0.0035	0.0019	0.0011
5000	Std	0.18	0.1	0.055	0.033	0.018	0.01	0.006	0.0032	0.0018	0.001	0.00057
5000	Max	0.92	0.51	0.28	0.16	0.09	0.051	0.029	0.019	0.009	0.0053	0.003
7500	Mean	0.28	0.16	0.09	0.05	0.027	0.016	0.009	0.0048	0.0027	0.0015	0.00089
7500	Std	0.15	0.081	0.047	0.028	0.014	0.0086	0.0047	0.0025	0.0015	0.0008	0.00047
7500	Max	0.88	0.39	0.26	0.16	0.073	0.045	0.024	0.013	0.0074	0.0039	0.0025
10,000	Mean	0.24	0.14	0.081	0.044	0.024	0.014	0.0076	0.0046	0.0024	0.0014	0.0008
10,000	Std	0.12	0.071	0.045	0.023	0.013	0.007	0.004	0.0023	0.0013	0.00074	0.00041
10,000	Max	0.66	0.37	0.23	0.11	0.068	0.036	0.02	0.013	0.0068	0.004	0.0021
20,000	Mean	0.17	0.098	0.057	0.032	0.018	0.0094	0.0056	0.003	0.0017	0.00097	0.00054
20,000	Std	0.096	0.051	0.03	0.016	0.0096	0.0053	0.0029	0.0015	0.00088	0.00052	0.00028
20,000	Max	0.53	0.27	0.17	0.089	0.047	0.029	0.014	0.0076	0.0043	0.0029	0.0013
50,000	Mean	0.11	0.063	0.036	0.02	0.011	0.006	0.0034	0.002	0.0011	0.00061	0.00034
50,000	Std	0.062	0.032	0.018	0.01	0.0056	0.0032	0.0017	0.001	0.00059	0.00032	0.00018
50,000	Max	0.32	0.17	0.1	0.054	0.031	0.018	0.0089	0.0052	0.0031	0.0016	0.00088

**Table 5:** Mean values, standard deviation, and maximum values of the mean error of the three emitters in the calculation of the reception point on the PSD surface using FDMA with IQ as a function of SNR and signal size for experimental results.

Signal Length (Samples)	Error (mm)	60 dB	66 dB
1000	Mean	0.011	0.0052
1000	Std	0.0058	0.0028
1000	Max	0.036	0.019
2000	Mean	0.0088	0.0042
2000	Std	0.0047	0.0022
2000	Max	0.031	0.015
5000	Mean	0.0057	0.0028
5000	Std	0.003	0.0014
5000	Max	0.019	0.0082
7500	Mean	0.0047	0.0023
7500	Std	0.0025	0.0012
7500	Max	0.014	0.0068
10,000	Mean	0.0041	0.002
10,000	Std	0.0022	0.0011
10,000	Max	0.012	0.0062
20,000	Mean	0.003	0.0014
20,000	Std	0.0016	0.00077
20,000	Max	0.0084	0.0038
50,000	Mean	0.0019	0.00094
50,000	Std	0.00098	0.00045
50,000	Max	0.005	0.0023

## Abbreviations

The following abbreviations are used in this manuscript:

AoA	Angle of Arrival
BPSK	Binary Phase-Shift Keying
BPF	Band Pass Filter
CDMA	Code Division Multiple Access
CRC	Cyclic Redundancy Check
DFT	Discrete Fourier Transform
ELDLL	Early Late Delay Lock Loop
FDMA	Frequency Division Multiple Access
FFT	Fast Fourier Transform
FoV	Field of View
IPS	Indoor Positioning System
IRED	Infrared
LOS	Line of Sight
LPF	Low Pass Filter
LPS	Local Positioning System
MAI	Multiple Access Interference
MP	Multipath
OFDMA	Orthogonal Frequency Division Multiplexing
PDoA	Phase Differential of Arrival
PIC	Parallel Interference Cancellation
PoA	Phase of Arrival
PN	Pseudo Random
PSD	Position Sensitive Device
RMS	Root Mean Square
RSS	Received Signal Strength
SDMA	Spatial Division Multiple Access
SIC	Successive Interference Cancellation
TDMA	Time Division Multiple Access
VLP	Visible Light Positioning system

## Appendix A. Noise Signal Generation

Since we work with electronic systems, there are always noise components. This noise can be noise from the receiver, the channel, or the transmitter itself. All the noises are added up to a single noise component. The noise considered was white Gaussian noise. To simulate different scenarios, the signal-to-noise ratio value SNR was used. Depending on the received signal and the SNR to be analyzed, a different noise signal was generated.

Therefore, the following strategy was followed when introducing noise to the simulations:

1. The signal received by each of the four channels of the PSD was obtained in the absence of noise.
2. The total signal power $P_{s}$ received by the PSD was calculated, that is, the sum of the four signals of the four channels of the PSD.
3. The noise power spectral density $N_{0}$ was calculated as:
  (A1) $$N_{0}=\frac{P_{s}}{10^{{\mathrm{SNR}}_{\mathrm{dBHz}}/10}}$$
4. From the bandwidth BW and $N_{0}$, the noise power was calculated:
  (A2) $$P_{n}=N_{0}\mathrm{BW}$$
5. For each channel of the PSD, white Gaussian noise was generated with a noise power of one quarter of the total power. The generated noise was calculated from the following expression
  (A3) $$\mathrm{noise}=\sqrt{\frac{P_{n}}{4}}\mathrm{randn}$$
  where randn is Matlab’s function for generating random numbers with a normal distribution.
6. Finally, each of the four noise signals was added with the signal of each of the four channels.

## Appendix B. Results Tables of the Behavior of the Different Techniques in Noisy Environments

This appendix shows the simulation results described in Section 4.1.

**Table A1:** Mean values, standard deviation, and maximum error values in the calculation of the impact point using FDMA with IQ with a 8-bit size.

Index	Error (mm)	57 dBHz	62 dBHz	67 dBHz	72 dBHz	77 dBHz	82 dBHz	87 dBHz	92 dBHz	97 dBHz	102 dBHz	107 dBHz
1	Mean	0.18	0.053	0.031	0.017	0.0097	0.0057	0.0031	0.0016	0.00098	0.00055	0.00031
1	Std	0.086	0.027	0.017	0.0096	0.005	0.0029	0.0016	0.0009	0.00051	0.00029	0.00015
1	Max	0.42	0.14	0.083	0.049	0.027	0.014	0.0073	0.006	0.0028	0.0013	0.00095
2	Mean	0.18	0.058	0.031	0.018	0.01	0.0051	0.003	0.0017	0.00093	0.00055	0.00029
2	Std	0.098	0.028	0.015	0.0086	0.0055	0.0027	0.0017	0.00089	0.00049	0.00024	0.00017
2	Max	0.5	0.14	0.078	0.037	0.03	0.015	0.0082	0.0045	0.0023	0.0013	0.00091
3	Mean	0.17	0.057	0.029	0.017	0.0097	0.0053	0.003	0.0016	0.001	0.00054	0.00031
3	Std	0.092	0.03	0.014	0.0082	0.005	0.003	0.0016	0.00088	0.00054	0.00027	0.00015
3	Max	0.44	0.15	0.072	0.041	0.03	0.018	0.0089	0.0044	0.0027	0.0013	0.00072
4	Mean	0.17	0.055	0.03	0.017	0.0097	0.0054	0.003	0.0017	0.00096	0.00052	0.00033
4	Std	0.09	0.029	0.016	0.0086	0.0054	0.0027	0.0015	0.00093	0.00055	0.00027	0.00017
4	Max	0.49	0.17	0.086	0.046	0.032	0.015	0.007	0.0049	0.0033	0.0014	0.0011
5	Mean	0.17	0.058	0.031	0.017	0.011	0.0056	0.0031	0.0019	0.001	0.00053	0.00033
5	Std	0.087	0.031	0.015	0.0082	0.0051	0.003	0.0017	0.00095	0.0005	0.00028	0.00017
5	Max	0.42	0.17	0.071	0.043	0.026	0.014	0.0077	0.0046	0.0027	0.0014	0.00094
6	Mean	0.17	0.056	0.032	0.017	0.011	0.0058	0.0035	0.0019	0.00093	0.00056	0.00032
6	Std	0.1	0.029	0.017	0.0088	0.006	0.0029	0.0017	0.00094	0.00045	0.0003	0.00018
6	Max	0.57	0.16	0.1	0.052	0.03	0.016	0.0089	0.0061	0.0023	0.0015	0.001
7	Mean	0.18	0.057	0.032	0.018	0.01	0.0054	0.0032	0.0018	0.001	0.00057	0.0003
7	Std	0.096	0.03	0.016	0.0092	0.0053	0.0027	0.0016	0.00088	0.00053	0.00033	0.00015
7	Max	0.58	0.17	0.093	0.054	0.027	0.013	0.0079	0.0044	0.0024	0.0016	0.00084
8	Mean	0.17	0.054	0.033	0.018	0.0099	0.0055	0.0032	0.0018	0.001	0.00058	0.0003
8	Std	0.097	0.029	0.016	0.0093	0.0051	0.0029	0.0017	0.001	0.0005	0.0003	0.00016
8	Max	0.48	0.17	0.076	0.054	0.028	0.015	0.0091	0.0048	0.0025	0.0015	0.00078
9	Mean	0.19	0.056	0.03	0.017	0.0094	0.0059	0.0033	0.0019	0.0011	0.00055	0.00035
9	Std	0.099	0.026	0.016	0.0099	0.0048	0.003	0.0018	0.00095	0.00056	0.00026	0.00018
9	Max	0.42	0.14	0.083	0.055	0.022	0.016	0.0087	0.0056	0.0027	0.0014	0.00089
10	Mean	0.19	0.056	0.031	0.018	0.01	0.0061	0.003	0.0018	0.0011	0.00056	0.00032
10	Std	0.096	0.031	0.016	0.0093	0.0056	0.0032	0.0015	0.00097	0.00058	0.0003	0.00017
10	Max	0.47	0.15	0.069	0.044	0.028	0.016	0.0088	0.0047	0.0027	0.0015	0.00085

**Table A2:** Mean values, standard deviation, and maximum error values in the calculation of the impact point using CDMA-BPSK with a 8-bit size.

Index	Error (mm)	57 dBHz	62 dBHz	67 dBHz	72 dBHz	77 dBHz	82 dBHz	87 dBHz	92 dBHz	97 dBHz	102 dBHz	107 dBHz
1	Mean	0.19	0.054	0.03	0.018	0.0099	0.0055	0.0031	0.0018	0.001	0.00054	0.00032
1	Std	0.094	0.029	0.016	0.0092	0.0053	0.0031	0.0017	0.00093	0.00052	0.0003	0.00018
1	Max	0.48	0.13	0.09	0.046	0.027	0.016	0.0076	0.0048	0.0025	0.0019	0.00089
2	Mean	0.17	0.058	0.032	0.019	0.01	0.0056	0.0034	0.0018	0.00096	0.00055	0.00032
2	Std	0.084	0.032	0.015	0.0091	0.0057	0.0029	0.0018	0.00089	0.00053	0.00032	0.00019
2	Max	0.43	0.17	0.077	0.039	0.026	0.015	0.0095	0.0043	0.0028	0.0015	0.00092
3	Mean	0.18	0.058	0.032	0.018	0.01	0.0057	0.0034	0.0017	0.00097	0.00055	0.00031
3	Std	0.091	0.029	0.016	0.0095	0.0054	0.0028	0.0016	0.00086	0.00053	0.00029	0.00016
3	Max	0.44	0.15	0.073	0.059	0.029	0.014	0.0085	0.0041	0.0023	0.0015	0.00097
4	Mean	0.18	0.054	0.033	0.018	0.011	0.0054	0.0033	0.0017	0.0011	0.00056	0.00033
4	Std	0.099	0.028	0.019	0.0096	0.005	0.0027	0.0016	0.00084	0.00054	0.00029	0.00017
4	Max	0.46	0.15	0.1	0.048	0.027	0.012	0.0077	0.0041	0.0029	0.0014	0.0009
5	Mean	0.19	0.06	0.031	0.018	0.01	0.0058	0.0032	0.0018	0.001	0.0006	0.00035
5	Std	0.098	0.032	0.017	0.0093	0.0052	0.0029	0.0017	0.00095	0.00057	0.00032	0.00018
5	Max	0.59	0.19	0.099	0.05	0.029	0.015	0.01	0.0057	0.0026	0.0015	0.00089
6	Mean	0.18	0.061	0.033	0.018	0.01	0.0062	0.0034	0.0019	0.001	0.00058	0.00034
6	Std	0.081	0.031	0.017	0.0091	0.0051	0.0029	0.0017	0.00095	0.00052	0.00033	0.00016
6	Max	0.44	0.18	0.11	0.042	0.025	0.019	0.0088	0.0047	0.0031	0.0015	0.00084
7	Mean	0.19	0.061	0.034	0.019	0.011	0.0057	0.0035	0.0019	0.001	0.00062	0.00034
7	Std	0.1	0.032	0.018	0.011	0.0055	0.003	0.0018	0.00092	0.00055	0.00031	0.00018
7	Max	0.48	0.18	0.085	0.05	0.03	0.016	0.011	0.0045	0.0032	0.0016	0.00088
8	Mean	0.18	0.061	0.035	0.019	0.011	0.0055	0.0035	0.0019	0.0011	0.0006	0.00033
8	Std	0.088	0.034	0.018	0.0095	0.0057	0.0028	0.0017	0.00098	0.00057	0.0003	0.00018
8	Max	0.46	0.18	0.1	0.063	0.03	0.015	0.0089	0.0045	0.0029	0.0014	0.00092
9	Mean	0.2	0.056	0.036	0.019	0.011	0.0061	0.0034	0.002	0.0011	0.00062	0.00035
9	Std	0.09	0.033	0.018	0.0094	0.0056	0.0032	0.0019	0.00095	0.00056	0.00032	0.0002
9	Max	0.43	0.17	0.1	0.058	0.038	0.017	0.009	0.0045	0.0029	0.0015	0.00089
10	Mean	0.2	0.059	0.033	0.02	0.011	0.0065	0.0035	0.002	0.0011	0.00061	0.00034
10	Std	0.1	0.028	0.017	0.01	0.0056	0.0034	0.0017	0.001	0.00057	0.0003	0.00019
10	Max	0.57	0.14	0.08	0.047	0.031	0.02	0.0094	0.0047	0.0032	0.0015	0.00098

**Table A3:** Mean values, standard deviation, and maximum error values in the calculation of the impact point using CDMA-BPSK with a 10-bit size.

Index	Error (mm)	57 dBHz	62 dBHz	67 dBHz	72 dBHz	77 dBHz	82 dBHz	87 dBHz	92 dBHz	97 dBHz	102 dBHz	107 dBHz
1	Mean	0.086	0.028	0.016	0.0088	0.0051	0.0028	0.0016	0.00094	0.00054	0.00028	0.00017
1	Std	0.047	0.014	0.008	0.0046	0.0026	0.0015	0.00079	0.00046	0.00025	0.00014	9.1 × $10^{-5}$
1	Max	0.23	0.079	0.038	0.022	0.013	0.0087	0.0047	0.0024	0.0012	0.00094	0.00045
2	Mean	0.092	0.026	0.015	0.0094	0.005	0.0029	0.0015	0.00091	0.0005	0.00029	0.00017
2	Std	0.045	0.014	0.0089	0.0053	0.0027	0.0014	0.00084	0.00047	0.00024	0.00015	7.9 × $10^{-5}$
2	Max	0.22	0.084	0.05	0.031	0.014	0.0083	0.0045	0.0022	0.0015	0.00092	0.00047
3	Mean	0.091	0.028	0.016	0.0093	0.0051	0.0029	0.0016	0.00095	0.00049	0.00029	0.00017
3	Std	0.045	0.014	0.0082	0.0045	0.0029	0.0015	0.00085	0.00049	0.00026	0.00015	8.7 × $10^{-5}$
3	Max	0.25	0.07	0.046	0.024	0.016	0.0072	0.0046	0.0024	0.0014	0.00076	0.00043
4	Mean	0.092	0.03	0.017	0.0093	0.0052	0.0028	0.0016	0.00088	0.00051	0.00027	0.00015
4	Std	0.045	0.016	0.009	0.005	0.0027	0.0015	0.00084	0.00045	0.00027	0.00015	7.7 × $10^{-5}$
4	Max	0.26	0.086	0.043	0.026	0.013	0.0079	0.0041	0.0019	0.0012	0.0009	0.00046
5	Mean	0.085	0.026	0.016	0.0095	0.005	0.0027	0.0016	0.00096	0.00051	0.00029	0.00017
5	Std	0.048	0.015	0.0087	0.0053	0.0026	0.0014	0.00093	0.00048	0.00027	0.00016	8.6 × $10^{-5}$
5	Max	0.24	0.079	0.046	0.027	0.013	0.0069	0.0051	0.0023	0.0014	0.0011	0.00041
6	Mean	0.092	0.029	0.017	0.0087	0.005	0.0028	0.0017	0.00094	0.00053	0.00029	0.00017
6	Std	0.046	0.016	0.0089	0.0051	0.0029	0.0016	0.00085	0.00048	0.00029	0.00016	8.8 × $10^{-5}$
6	Max	0.27	0.087	0.042	0.024	0.015	0.0086	0.0041	0.0022	0.0013	0.0007	0.00043
7	Mean	0.091	0.03	0.016	0.0091	0.0056	0.0028	0.0016	0.00089	0.0005	0.0003	0.00017
7	Std	0.049	0.015	0.009	0.005	0.0027	0.0013	0.0008	0.00046	0.00026	0.00014	8.5 × $10^{-5}$
7	Max	0.27	0.083	0.049	0.025	0.016	0.0079	0.0043	0.0027	0.0014	0.00091	0.00048
8	Mean	0.088	0.029	0.018	0.0092	0.0051	0.003	0.0018	0.00086	0.00049	0.00029	0.00017
8	Std	0.042	0.016	0.0089	0.0048	0.0029	0.0016	0.00091	0.00046	0.00027	0.00015	8.6 × $10^{-5}$
8	Max	0.24	0.069	0.043	0.027	0.013	0.0071	0.0042	0.0022	0.0013	0.00074	0.00041
9	Mean	0.094	0.031	0.018	0.0097	0.0053	0.0031	0.0017	0.0009	0.0005	0.0003	0.00017
9	Std	0.047	0.018	0.0096	0.005	0.0027	0.0016	0.00087	0.00053	0.00025	0.00016	9.6 × $10^{-5}$
9	Max	0.23	0.092	0.051	0.032	0.015	0.0078	0.0047	0.0027	0.0014	0.00075	0.00051
10	Mean	0.098	0.03	0.017	0.01	0.0053	0.003	0.0018	0.00098	0.00054	0.00029	0.00017
10	Std	0.05	0.016	0.0094	0.0047	0.0026	0.0017	0.00096	0.00048	0.00027	0.00015	8.7 × $10^{-5}$
10	Max	0.26	0.072	0.046	0.024	0.012	0.0096	0.0048	0.0021	0.0015	0.0008	0.00047

**Table A4:** Mean values, standard deviation, and maximum error values in the calculation of the impact point using FDMA-BPF with a 8-bit size.

Index	Error (mm)	57 dBHz	62 dBHz	67 dBHz	72 dBHz	77 dBHz	82 dBHz	87 dBHz	92 dBHz	97 dBHz	102 dBHz	107 dBHz
1	Mean	0.19	0.061	0.033	0.018	0.011	0.0058	0.0034	0.0018	0.00096	0.00058	0.00034
1	Std	0.1	0.032	0.018	0.0096	0.0058	0.0033	0.0018	0.00091	0.00052	0.0003	0.00018
1	Max	0.5	0.15	0.1	0.053	0.028	0.018	0.0086	0.0047	0.0031	0.0016	0.00092
2	Mean	0.19	0.058	0.034	0.02	0.011	0.0061	0.0034	0.002	0.0011	0.0006	0.00035
2	Std	0.097	0.028	0.017	0.01	0.0061	0.0035	0.0018	0.001	0.00059	0.00033	0.00019
2	Max	0.57	0.14	0.083	0.053	0.03	0.016	0.011	0.0065	0.0027	0.0015	0.00099
3	Mean	0.19	0.06	0.032	0.019	0.011	0.006	0.0036	0.0019	0.0012	0.00064	0.00035
3	Std	0.097	0.031	0.018	0.01	0.0056	0.0032	0.0018	0.00099	0.00062	0.00032	0.00019
3	Max	0.49	0.16	0.088	0.048	0.027	0.015	0.01	0.0049	0.003	0.0016	0.00097
4	Mean	0.2	0.063	0.036	0.02	0.011	0.0057	0.0034	0.0019	0.001	0.00062	0.00037
4	Std	0.095	0.034	0.02	0.012	0.0061	0.0034	0.0018	0.00098	0.00058	0.00034	0.00021
4	Max	0.46	0.19	0.11	0.07	0.03	0.016	0.0099	0.0049	0.0028	0.0018	0.00096
5	Mean	0.2	0.061	0.035	0.019	0.012	0.0059	0.0036	0.002	0.0011	0.00063	0.00034
5	Std	0.1	0.033	0.018	0.01	0.0055	0.003	0.0019	0.001	0.00059	0.00032	0.00018
5	Max	0.53	0.16	0.088	0.051	0.03	0.015	0.011	0.005	0.0028	0.0015	0.00086
6	Mean	0.21	0.062	0.035	0.02	0.011	0.0061	0.0036	0.0019	0.0011	0.00067	0.00034
6	Std	0.11	0.033	0.019	0.0087	0.0055	0.0031	0.0018	0.001	0.00059	0.00036	0.00018
6	Max	0.49	0.17	0.097	0.043	0.028	0.016	0.0086	0.0047	0.0027	0.002	0.00097
7	Mean	0.21	0.066	0.036	0.02	0.011	0.0061	0.0037	0.002	0.0011	0.00061	0.00036
7	Std	0.11	0.034	0.019	0.011	0.0057	0.0032	0.0018	0.001	0.00057	0.00034	0.00019
7	Max	0.54	0.16	0.094	0.058	0.028	0.017	0.0096	0.0052	0.0027	0.0019	0.00087
8	Mean	0.2	0.064	0.034	0.02	0.011	0.0058	0.0037	0.0019	0.0012	0.00062	0.00033
8	Std	0.11	0.033	0.017	0.01	0.0058	0.0032	0.0019	0.00096	0.0006	0.00033	0.00018
8	Max	0.65	0.19	0.094	0.052	0.026	0.016	0.0087	0.0048	0.003	0.0019	0.00083
9	Mean	0.19	0.067	0.036	0.021	0.011	0.0064	0.0036	0.002	0.0011	0.00063	0.00037
9	Std	0.097	0.034	0.019	0.011	0.0063	0.0038	0.0019	0.0011	0.00061	0.00033	0.0002
9	Max	0.47	0.19	0.11	0.051	0.037	0.021	0.01	0.0053	0.0029	0.0017	0.001
10	Mean	0.21	0.065	0.034	0.02	0.011	0.0062	0.0036	0.0019	0.0011	0.00065	0.00038
10	Std	0.11	0.033	0.02	0.011	0.0058	0.0034	0.0019	0.001	0.00061	0.00033	0.00018
10	Max	0.54	0.21	0.12	0.069	0.028	0.018	0.0093	0.0053	0.0036	0.002	0.00099

**Table A5:** Mean values, standard deviation, and maximum error values in the calculation of the impact point using FDMA-BPF with a 10-bit size.

Index	Error (mm)	57 dBHz	62 dBHz	67 dBHz	72 dBHz	77 dBHz	82 dBHz	87 dBHz	92 dBHz	97 dBHz	102 dBHz	107 dBHz
1	Mean	0.083	0.03	0.016	0.0089	0.0048	0.0028	0.0016	0.0009	0.00051	0.00029	0.00016
1	Std	0.044	0.016	0.0082	0.0049	0.0026	0.0014	0.00085	0.00049	0.00027	0.00014	8.7 × $10^{-5}$
1	Max	0.2	0.079	0.054	0.023	0.013	0.0069	0.0047	0.0026	0.0013	0.00094	0.0005
2	Mean	0.094	0.029	0.016	0.0091	0.0051	0.0027	0.0016	0.00093	0.0005	0.00027	0.00016
2	Std	0.048	0.014	0.0084	0.0045	0.0026	0.0014	0.0009	0.0005	0.00023	0.00012	8.2 × $10^{-5}$
2	Max	0.25	0.072	0.052	0.021	0.014	0.0067	0.0047	0.0031	0.0012	0.00065	0.00044
3	Mean	0.088	0.029	0.016	0.0094	0.0047	0.0028	0.0016	0.00093	0.0005	0.0003	0.00016
3	Std	0.044	0.017	0.0084	0.0044	0.0028	0.0014	0.00085	0.00045	0.00026	0.00016	8.6 × $10^{-5}$
3	Max	0.2	0.077	0.043	0.023	0.014	0.0075	0.0041	0.0024	0.0013	0.00084	0.00041
4	Mean	0.098	0.027	0.016	0.0097	0.0051	0.003	0.0017	0.00089	0.0005	0.00031	0.00015
4	Std	0.051	0.014	0.0082	0.0051	0.0026	0.0015	0.00085	0.00045	0.00026	0.00014	7.8 × $10^{-5}$
4	Max	0.3	0.075	0.039	0.027	0.011	0.0081	0.0052	0.0025	0.0013	0.00082	0.00043
5	Mean	0.097	0.029	0.016	0.0095	0.005	0.003	0.0016	0.00087	0.00049	0.00028	0.00016
5	Std	0.049	0.016	0.0081	0.005	0.0028	0.0015	0.00084	0.00048	0.00026	0.00014	8.2 × $10^{-5}$
5	Max	0.26	0.079	0.039	0.023	0.016	0.007	0.0045	0.0025	0.0015	0.00069	0.00046
6	Mean	0.096	0.031	0.016	0.0091	0.0053	0.003	0.0016	0.00092	0.00052	0.0003	0.00017
6	Std	0.05	0.017	0.0085	0.0049	0.0028	0.0015	0.00084	0.00049	0.00025	0.00014	9 × $10^{-5}$
6	Max	0.24	0.093	0.053	0.022	0.013	0.0074	0.0042	0.0024	0.0013	0.00078	0.00045
7	Mean	0.096	0.028	0.017	0.0096	0.0054	0.003	0.0016	0.00096	0.00052	0.00029	0.00015
7	Std	0.05	0.015	0.0097	0.005	0.0027	0.0015	0.00097	0.00048	0.00027	0.00015	8.2 × $10^{-5}$
7	Max	0.27	0.084	0.046	0.027	0.015	0.0073	0.0057	0.0024	0.0016	0.00077	0.00038
8	Mean	0.091	0.029	0.018	0.0091	0.0052	0.0031	0.0016	0.00095	0.00051	0.00029	0.00017
8	Std	0.052	0.016	0.0093	0.005	0.0026	0.0015	0.00082	0.00045	0.00026	0.00016	9 × $10^{-5}$
8	Max	0.25	0.081	0.057	0.027	0.012	0.0075	0.004	0.0024	0.0013	0.00077	0.00044
9	Mean	0.1	0.03	0.018	0.0094	0.0053	0.0029	0.0017	0.00096	0.00054	0.0003	0.00017
9	Std	0.054	0.016	0.0092	0.005	0.0025	0.0015	0.00089	0.00049	0.00031	0.00016	8.5 × $10^{-5}$
9	Max	0.26	0.083	0.045	0.025	0.012	0.0089	0.004	0.0028	0.0017	0.00075	0.0004
10	Mean	0.12	0.031	0.018	0.0093	0.0053	0.0027	0.0018	0.001	0.00052	0.00029	0.00018
10	Std	0.063	0.016	0.0093	0.0047	0.0028	0.0016	0.001	0.00051	0.00028	0.00013	9.3 × $10^{-5}$
10	Max	0.29	0.068	0.051	0.022	0.017	0.0076	0.0048	0.0025	0.0014	0.00078	0.00047

**Table A6:** Mean values, standard deviation, and maximum error values in the calculation of the impact point using FDMA-FFT with a 8-bit size.

Index	Error (mm)	57 dBHz	62 dBHz	67 dBHz	72 dBHz	77 dBHz	82 dBHz	87 dBHz	92 dBHz	97 dBHz	102 dBHz	107 dBHz
1	Mean	0.17	0.057	0.033	0.018	0.0096	0.0057	0.003	0.0019	0.00094	0.00061	0.00029
1	Std	0.097	0.028	0.017	0.0098	0.0053	0.003	0.0016	0.00095	0.00053	0.00032	0.00017
1	Max	0.56	0.14	0.075	0.045	0.025	0.015	0.0089	0.0055	0.0032	0.0017	0.0011
2	Mean	0.18	0.056	0.032	0.018	0.0096	0.0058	0.0032	0.0018	0.0011	0.00055	0.0003
2	Std	0.095	0.029	0.018	0.0092	0.005	0.0031	0.0016	0.00099	0.00051	0.00027	0.00016
2	Max	0.57	0.16	0.12	0.042	0.024	0.016	0.0076	0.0048	0.0025	0.0012	0.00073
3	Mean	0.17	0.054	0.032	0.018	0.0094	0.0054	0.003	0.0017	0.00097	0.00056	0.00032
3	Std	0.089	0.029	0.018	0.009	0.0052	0.0028	0.0016	0.00093	0.00053	0.00029	0.00017
3	Max	0.42	0.16	0.092	0.044	0.027	0.015	0.0083	0.0053	0.003	0.0014	0.0011
4	Mean	0.18	0.055	0.03	0.018	0.01	0.0058	0.0033	0.0018	0.001	0.00054	0.00033
4	Std	0.092	0.028	0.016	0.0094	0.0046	0.0031	0.0015	0.0009	0.00056	0.00027	0.00016
4	Max	0.48	0.14	0.09	0.054	0.025	0.016	0.0083	0.0043	0.0027	0.0014	0.00085
5	Mean	0.18	0.058	0.033	0.018	0.0097	0.0059	0.0031	0.0018	0.00097	0.00058	0.00032
5	Std	0.09	0.028	0.017	0.0094	0.0053	0.003	0.0017	0.001	0.00056	0.00033	0.00016
5	Max	0.46	0.16	0.08	0.047	0.028	0.017	0.0074	0.0051	0.0028	0.0015	0.0008
6	Mean	0.17	0.059	0.033	0.018	0.011	0.006	0.0033	0.0019	0.00093	0.00057	0.00034
6	Std	0.085	0.033	0.016	0.0098	0.0055	0.0031	0.0017	0.00093	0.00048	0.00029	0.00017
6	Max	0.36	0.17	0.075	0.048	0.03	0.016	0.0089	0.0051	0.0026	0.0014	0.00096
7	Mean	0.18	0.059	0.034	0.02	0.01	0.0056	0.0032	0.0018	0.0011	0.00058	0.00033
7	Std	0.091	0.033	0.019	0.0095	0.0061	0.003	0.0017	0.00097	0.00055	0.00031	0.00017
7	Max	0.42	0.19	0.084	0.048	0.029	0.016	0.0088	0.0046	0.0032	0.0015	0.00097
8	Mean	0.18	0.061	0.034	0.018	0.011	0.0059	0.0034	0.0019	0.0011	0.00057	0.00032
8	Std	0.096	0.034	0.018	0.0089	0.0055	0.0032	0.0016	0.00097	0.00056	0.00032	0.00018
8	Max	0.48	0.16	0.087	0.049	0.028	0.017	0.0081	0.0046	0.0026	0.0014	0.00087
9	Mean	0.18	0.061	0.035	0.019	0.01	0.0055	0.0033	0.0019	0.001	0.00062	0.00032
9	Std	0.091	0.032	0.016	0.01	0.0054	0.0027	0.0017	0.0011	0.0005	0.00032	0.00016
9	Max	0.5	0.16	0.095	0.057	0.027	0.014	0.0078	0.0055	0.0024	0.0016	0.00071
10	Mean	0.2	0.058	0.033	0.018	0.011	0.0061	0.0033	0.0017	0.0011	0.00058	0.00035
10	Std	0.11	0.031	0.017	0.01	0.0061	0.0032	0.0018	0.00093	0.00056	0.0003	0.00017
10	Max	0.57	0.15	0.075	0.051	0.031	0.016	0.009	0.0051	0.0027	0.0014	0.00086

**Table A7:** Mean values, standard deviation, and maximum error values in the calculation of the impact point using FDMA-FFT with a 10-bit size.

Index	Error (mm)	57 dBHz	62 dBHz	67 dBHz	72 dBHz	77 dBHz	82 dBHz	87 dBHz	92 dBHz	97 dBHz	102 dBHz	107 dBHz
1	Mean	0.089	0.028	0.016	0.0086	0.0052	0.0028	0.0015	0.0009	0.00048	0.00029	0.00015
1	Std	0.044	0.015	0.0081	0.0042	0.0026	0.0014	0.00088	0.00045	0.00026	0.00014	8 × $10^{-5}$
1	Max	0.24	0.078	0.045	0.021	0.014	0.0064	0.0045	0.0022	0.0012	0.00073	0.00039
2	Mean	0.086	0.029	0.016	0.0089	0.005	0.0027	0.0015	0.00091	0.00051	0.00027	0.00017
2	Std	0.048	0.015	0.0084	0.0046	0.0027	0.0014	0.00081	0.00047	0.00029	0.00014	8.7 × $10^{-5}$
2	Max	0.26	0.081	0.041	0.03	0.014	0.0084	0.0043	0.0024	0.0016	0.00067	0.00043
3	Mean	0.089	0.026	0.015	0.0089	0.0047	0.0027	0.0017	0.00087	0.00053	0.00029	0.00016
3	Std	0.043	0.015	0.0079	0.0046	0.0026	0.0014	0.00081	0.00046	0.00026	0.00015	8 × $10^{-5}$
3	Max	0.22	0.076	0.04	0.023	0.012	0.0072	0.004	0.0023	0.0015	0.00087	0.00049
4	Mean	0.089	0.03	0.016	0.0096	0.0047	0.0028	0.0016	0.00089	0.00051	0.0003	0.00017
4	Std	0.045	0.017	0.0078	0.0051	0.0026	0.0015	0.00079	0.00047	0.00025	0.00016	8.3 × $10^{-5}$
4	Max	0.22	0.097	0.036	0.024	0.013	0.0093	0.004	0.0025	0.0013	0.00084	0.00052
5	Mean	0.091	0.03	0.016	0.0082	0.005	0.0027	0.0016	0.00091	0.0005	0.00029	0.00016
5	Std	0.051	0.017	0.0086	0.0044	0.0027	0.0015	0.00081	0.00049	0.00027	0.00015	8.6 × $10^{-5}$
5	Max	0.23	0.075	0.049	0.029	0.014	0.0095	0.0041	0.0025	0.0015	0.00073	0.00039
6	Mean	0.089	0.029	0.015	0.009	0.005	0.0029	0.0017	0.0009	0.00054	0.00028	0.00016
6	Std	0.048	0.016	0.0082	0.0048	0.0024	0.0015	0.00083	0.00048	0.00028	0.00014	8.9 × $10^{-5}$
6	Max	0.22	0.087	0.042	0.023	0.012	0.0069	0.0045	0.0025	0.0016	0.00078	0.00046
7	Mean	0.093	0.029	0.016	0.0096	0.0055	0.0028	0.0016	0.0009	0.0005	0.00029	0.00016
7	Std	0.05	0.014	0.0083	0.0053	0.0028	0.0015	0.00093	0.00044	0.00026	0.00015	8.1 × $10^{-5}$
7	Max	0.24	0.081	0.043	0.026	0.014	0.0072	0.005	0.0023	0.0013	0.00077	0.00042
8	Mean	0.087	0.028	0.015	0.0096	0.0052	0.0029	0.0016	0.00089	0.00048	0.0003	0.00016
8	Std	0.046	0.014	0.0083	0.0055	0.0027	0.0016	0.00084	0.00048	0.00024	0.00015	8.3 × $10^{-5}$
8	Max	0.24	0.072	0.041	0.027	0.015	0.0088	0.0047	0.0026	0.0012	0.00081	0.00046
9	Mean	0.094	0.028	0.017	0.0095	0.0054	0.0028	0.0017	0.00089	0.00054	0.00029	0.00017
9	Std	0.049	0.015	0.0084	0.005	0.0028	0.0015	0.00088	0.0005	0.0003	0.00015	9.1 × $10^{-5}$
9	Max	0.28	0.075	0.043	0.024	0.014	0.0075	0.0054	0.0027	0.0015	0.00081	0.00047
10	Mean	0.1	0.029	0.018	0.0093	0.0055	0.0028	0.0017	0.001	0.00053	0.00027	0.00016
10	Std	0.052	0.016	0.0086	0.0049	0.0027	0.0015	0.00084	0.00049	0.00025	0.00015	8.8 × $10^{-5}$
10	Max	0.26	0.071	0.045	0.022	0.014	0.0082	0.004	0.0024	0.0014	0.00081	0.00053
